# Delayed death in the malaria parasite *Plasmodium falciparum* is caused by disruption of prenylation-dependent intracellular trafficking

**DOI:** 10.1371/journal.pbio.3000376

**Published:** 2019-07-18

**Authors:** Kit Kennedy, Simon A. Cobbold, Eric Hanssen, Jakob Birnbaum, Natalie J. Spillman, Emma McHugh, Hannah Brown, Leann Tilley, Tobias Spielmann, Malcolm J. McConville, Stuart A. Ralph

**Affiliations:** 1 Department of Biochemistry and Molecular Biology, Bio21 Molecular Science and Biotechnology Institute, The University of Melbourne, Victoria, Australia; 2 Advanced Microscopy Facility, Bio21 Molecular Science and Biotechnology Institute, Victoria, Australia; 3 Molecular Biology and Immunology Section, Bernhard Nocht Institute for Tropical Medicine, Hamburg, Germany; University of South Florida, UNITED STATES

## Abstract

Apicomplexan parasites possess a plastid organelle called the apicoplast. Inhibitors that selectively target apicoplast housekeeping functions, including DNA replication and protein translation, are lethal for the parasite, and several (doxycycline, clindamycin, and azithromycin) are in clinical use as antimalarials. A major limitation of such drugs is that treated parasites only arrest one intraerythrocytic development cycle (approximately 48 hours) after treatment commences, a phenotype known as the ‘delayed death’ effect. The molecular basis of delayed death is a long-standing mystery in parasitology, and establishing the mechanism would aid rational clinical implementation of apicoplast-targeted drugs. Parasites undergoing delayed death transmit defective apicoplasts to their daughter cells and cannot produce the sole, blood-stage essential metabolic product of the apicoplast: the isoprenoid precursor isopentenyl-pyrophosphate. How the isoprenoid precursor depletion kills the parasite remains unknown. We investigated the requirements for the range of isoprenoids in the human malaria parasite *Plasmodium falciparum* and characterised the molecular and morphological phenotype of parasites experiencing delayed death. Metabolomic profiling reveals disruption of digestive vacuole function in the absence of apicoplast derived isoprenoids. Three-dimensional electron microscopy reveals digestive vacuole fragmentation and the accumulation of cytostomal invaginations, characteristics common in digestive vacuole disruption. We show that digestive vacuole disruption results from a defect in the trafficking of vesicles to the digestive vacuole. The loss of prenylation of vesicular trafficking proteins abrogates their membrane attachment and function and prevents the parasite from feeding. Our data show that the proximate cause of delayed death is an interruption of protein prenylation and consequent cellular trafficking defects.

## Abstract 2

After treatment with drugs that target apicoplast functions, malaria parasites are initially superficially healthy and go on to infect new erythrocytes. This cell biology study shows that the parasites subsequently die in their second cycle due to trafficking defects caused by depletion of prenyl groups.

## Introduction

The apicoplast, the plastid of the malaria parasite (*Plasmodium* spp.), is an essential and druggable organelle. The apicoplast is found in most apicomplexan parasites, with *Cryptosporidium* spp. being a notable exception [1]. The apicoplast is believed to be homologous to the chloroplast of dinoflagellates, being acquired by the common ancestor of Apicomplexa and Dinozoa through the secondary endosymbiosis of an ancient photosynthetic red alga [2,3]. The algal plastid has been retained in Apicomplexa despite losing its capacity for photosynthesis, which was lost during the transition to obligate parasitism. The apicoplast retains a 35-kb genome, originally derived from the cyanobacterial ancestor of all plastids, and expresses a small repertoire of gene products essential for the upkeep and replication of the organelle. The majority of the endosymbiont’s genes, required for the anabolic productivity of the apicoplast, have been transferred to the nucleus. These nuclear-encoded apicoplast proteins require a bipartite signal peptide to correctly traffic back to and translocate through the organelle’s four membranes.

A consequence of acquiring the plastid is that *Plasmodium* spp., as well as closely related human disease–causing Apicomplexans like *Toxoplasma gondii* maintain druggable prokaryotic-like mechanisms and pathways [4,5]. The apicoplast’s prokaryotic-like translation apparatus has been an especially well-exploited antiparasitic drug target [6–9]. Many antibiotics that chemically inhibit either the 30S or 50S bacterial ribosome, such as doxycycline and clindamycin, have been repurposed to treat both malaria and toxoplasmosis [10,11]. However, a defining characteristic for known inhibitors of apicoplast protein translation or genome replication is a curious chemotherapeutic effect referred to as delayed death.

Drugs targeting plastid maintenance cause no growth defect within the initial 48-hour intraerythrocytic development cycle (IDC). Rather, parasites continue to grow and complete schizogony. Daughter merozoites segment, egress, and invade a new host red blood cell (RBC), in which they continue to develop from ring-stage parasites to trophozoites. It is only later in this second IDC that the effect of the drug manifests and the treated parasites lethally arrest. Seemingly, parasites treated with drugs that target plastid maintenance acquire a defect in their apicoplast (first IDC) that becomes lethal when transmitted to their progeny (second IDC). This is readily observed as defective apicoplast protein import and organelle segmentation and a reduction in genome number in the second but not first IDC following treatment [4,7,12]. An effect comparable to delayed death is also observed in liver-stage *P*. *berghei* parasites [13]. Early liver-stage parasites treated with apicoplast-targeting antibiotics show no apparent defect during the intracellular expansion and division but fail to segregate the apicoplast properly and produce merosomes that do not subsequently establish a blood-stage infection [13]. A delayed-death phenotype is also observed for intracellular tachyzoites of *T*. *gondii*, in which chemical or genetic inhibition of apicoplast function or division is lethal only in the progeny of treated parasites [14–16]. Delayed death is an obstacle to development of some drugs targeting the apicoplast as stand-alone malaria treatments, although such compounds can be used in combination or as prophylactic drugs.

The apicoplast houses multiple anabolic pathways to synthesise metabolites for the growing parasite. Pathways include biosynthetic networks for type II fatty acid synthesis (FASII), iron–sulphur clusters (FE–S), lipoic acids, and haem, as well as a 2-C-methyl-D-erythritol 4-phosphate/1-deoxy-D-xylulose 5-phosphate (MEP/DOXP) pathway for isoprenoid biosynthesis [17]. Of these, Yeh and DeRisi [18] showed that the exogenous supply of the isoprenoid precursor isopentenyl pyrophosphate (IPP) was sufficient to rescue inhibition of apicoplast protein translation and, indeed, even complete ablation of the apicoplast in intraerythrocytic culture of *P*. *falciparum*. This indicates that isoprenoids are the sole essential metabolite supplied by the apicoplast to the rest of the cell during *P*. *falciparum* blood stages. While IPP rescue is not experimentally possible in *Toxoplasma*, recent data from Amberg-Johnson and Yeh [19] indicate that delayed death in *T*. *gondii* also depends on IPP availability. Curiously, whereas inhibition of apicoplast housekeeping leads to delayed death, chemical or genetic ablation of the MEP/DOXP pathway in *Plasmodium* or *Toxoplasma* parasites leads to immediate death [20–23]. These data suggest that while depletion of isoprenoids is immediately deleterious, delayed-death parasites either harbour a finite isoprenoid reservoir or can only temporarily continue to synthesise isoprenoids, likely using residual enzymes of the MEP/DOXP that were imported into the apicoplast in the first IDC. For delayed death in *T*. *gondii*, parasites lacking an apicoplast are able to survive by sharing metabolites between multiple parasites within a single parasitophorous vacuole [24], provided one individual retains an apicoplast [14].

We hypothesise that delayed death is the consequence of isoprenoid fatigue: parasites are eventually depleted of available isoprenoids but are unable to synthesise new precursors de novo. However, the individual essential isoprenoid products, the essential roles that these isoprenoids play, and how isoprenoid fatigue fatally perturbs molecular processes in *Plasmodium* spp. remain to be determined. IPP produced by the apicoplast likely generates a diverse collection of higher isoprenoids, which in *Plasmodium* spp. include 1) prenyl groups for protein modification, 2) the isoprene side chain of ubiquinone, and 3) dolichols for glycosylphosphatidylinositol (GPI) biosynthesis [25]. In this study, we have investigated the essentiality of different isoprenoid products by uncoupling the contribution that prenyl groups, ubiquinone, and dolichols have on delayed death. We have also characterised the effects of delayed-death inhibitors on parasite metabolism and cellular processes for the first time. We determined that the proximate cause of delayed death is disruption of protein prenylation and consequent cellular trafficking defects that culminate in arrested parasite growth. We find that parasites undergoing delayed death exhibited disrupted prenylation of proteins required for haemoglobin uptake and the biogenesis of the digestive vacuole (DV). We further demonstrate defective localisation of prenylated trafficking mediators, defective uptake of haemoglobin, and a disrupted DV in these parasites. Lastly, delineating the temporal contribution of prenylation from ubiquinone and dolichol biosynthesis to parasite development strongly indicates that disruption of prenylation is the proximate cause of delayed death.

## Results

### Metabolic perturbation of indolmycin-treated parasites during delayed death

**Fig 1:**
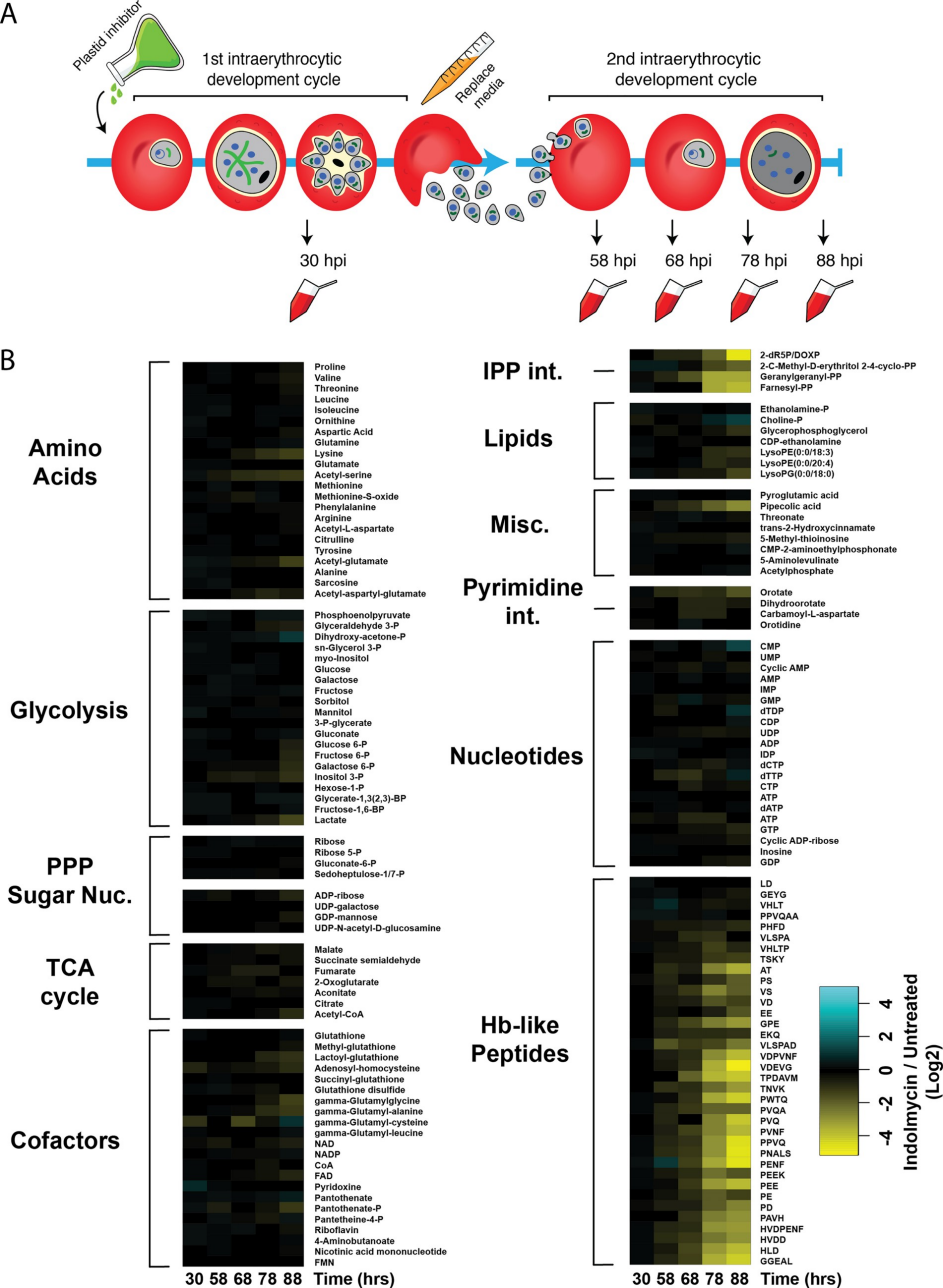
Untargeted metabolomic analysis of delayed death-parasites indicates isoprenoid defect and decreased haemoglobin turnover. (A) Schematic of delayed death in *P*. *falciparum*. During the first IDC, parasites develop over approximately 48 hours from the ring-stage into a metabolically active trophozoite, before dividing into multiple daughter merozoites by schizogony. Parasites treated with an apicoplast inhibitor during this first IDC successfully complete schizogony, but the daughter merozoites inherit a defective apicoplast. The lethal effect of the drug manifests only after invasion of a new host cell, in which parasites fail to complete a second IDC. Delayed-death parasites in this study were sampled for metabolomic analysis by LC-MS at the indicated time intervals post drug administration. (B) Heat map summarising the metabolic effect of the delayed-death inhibitor indolmycin (50 μM) on *P*. *falciparum-*infected RBC cultures. Total metabolite pools (measured as ion counts) across two IDCs were determined relative to untreated controls. Samples were collected for analysis at 30, 58, 68, 78, and 88 hours post drug administration, equivalent to 30 hpi in the first IDC and then 14, 24, 34, and 44 hpi in the second IDC. Changes in total metabolite pools are expressed as log2 ratios of indolmycin-treated cultures compared to untreated controls. Data are presented as the means of three independent experiments. See S1 Data for raw metabolomics data underlying heat map. hpi, hours post invasion; IDC, intraerythrocytic developmental cycle; LC-MS, liquid chromatography mass spectrometry; RBC, red blood cell.

We sought to investigate the metabolomic consequences of delayed death using specific inhibition of apicoplast translation. The inhibitor with the clearest direct evidence for an unambiguous apicoplast target is indolmycin, a specific inhibitor of the apicoplast tryptophanyl-tRNA synthetase (TrpRS^api^) in *P*. *falciparum* [26]. Indolmycin inhibits TrpRS^api^ activity in vitro, stops protein translation in the apicoplast, and causes delayed death in *P*. *falciparum* that can be rescued by IPP supplementation [26]. To investigate the metabolic consequence of delayed death on *P*. *falciparum*, infected RBCs (iRBCs) were treated with indolmycin at a concentration that causes no death in the first IDC but complete death in the second IDC (equivalent to 50× the 96-hour EC_50_ = 50 μM), and samples were collected at regular time intervals during the first and second IDC following treatment for analysis by liquid chromatography mass spectrometry (LC-MS) (Fig 1A).

The relative abundance (indolmycin treated/untreated) of the 159 metabolites detected in the time series is summarised in Fig 1B. Indolmycin treatment did not cause metabolic perturbation during the first IDC following treatment (30-hour time point). During the second IDC following drug treatment, the relative abundance of the isoprenoid biosynthetic intermediates 2-C-methyl-erythritol-2,4-cyclodiphosphate and DOXP, and the downstream isoprenoid species farnesyl pyrophosphate (FPP) and geranylgeranyl pyrophosphate (GGPP) progressively decreased in the indolmycin-treated parasites. The perturbation to isoprenoid biosynthesis is consistent with parasites possessing a defective apicoplast that cannot make new isoprenoids.

Few other metabolic changes were observed between indolmycin-treated and untreated parasites during the second IDC following drug treatment, with the exception of several haemoglobin-like peptides decreasing in abundance at 58 hours (equivalent to 14 hours post invasion [hpi] in the second IDC) and beyond (Fig 1B). This perturbation suggested that catabolism of RBC-derived haemoglobin may be compromised during indolmycin-induced delayed death. Diminished haemoglobin metabolism might be caused either by aberrant proteolysis in the parasite DV or by disrupted uptake/trafficking of haemoglobin to the DV. Given the essential role of prenylated proteins in vesicular trafficking and membrane fusion [27–29], it follows that decreased abundance of the prenyl precursors FPP and GGPP (Fig 1B) could disrupt the function of proteins involved in haemoglobin metabolism; below, we show that this is indeed the case.

### Protein prenylation is disrupted during delayed death

**Fig 2:**
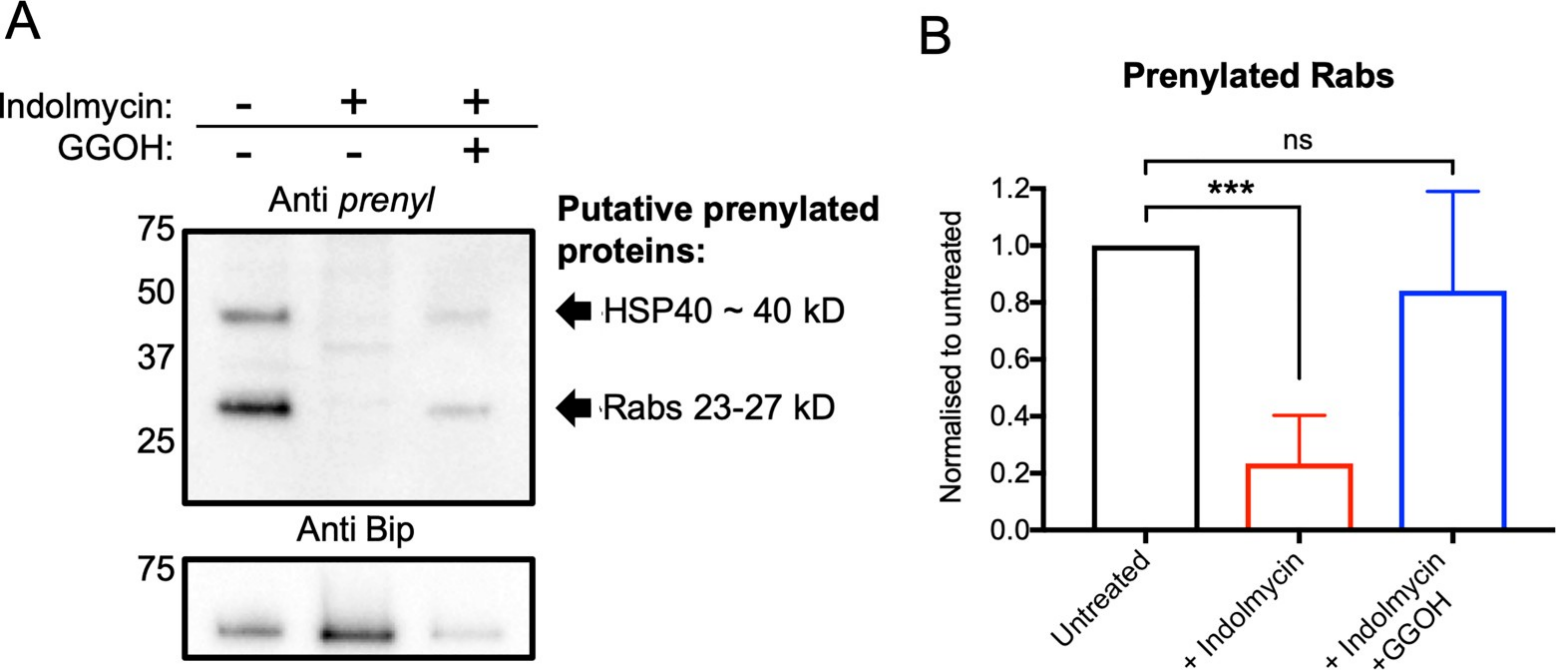
Apicoplast inhibitors decrease global parasite prenylation in the second IDC after treatment. (A) Immunoblot analysis of parasites treated with indolmycin (50 μM) with or without polyprenol rescue (5 μM GGOH), as indicated. Parasite lysates were collected after saponin isolation during their second IDC, and immunoblots were probed using anti-farnesyl (1:2,000) to analyse global parasite prenylation. Prenylated species are annotated as putative Rabs (23–27 kD) or HSP40 (40 kD) (assignment based on published *P*. *falciparum* prenylomes) [27,28]. Anti-Bip (1:1,000) were used as a loading control with an expected size of 65 kD. Immunoblot is representative of four independent experiments. (B) Quantification of the 27 kD Prenylated Rabs, normalised to average density of untreated. Data are presented as the means of four independent experiments ± SD. ***P < 0.001, two-tailed Student *t* test. See S2 Data for numerical data underlying figure. GGOH, geranylgeraniol; IDC, intraerythrocytic developmental cycle; ns, not significant.

To determine if the decreased abundance of the higher isoprenoid species and prenylation substrates FPP and GGPP detected in the metabolomics study corresponds to disruption of cellular protein prenylation, we used a commercial anti-farnesyl antibody, which is cross-reactive to proteins with a farnesyl or geranylgeranyl modification. iRBCs were treated with 50 μM indolmycin for 30 hours and, cell lysates were collected at 72–78 hours during their second IDC following treatment (equivalent to 28–34 hpi in the second IDC). In untreated (DMSO only) parasite lysates, two prominent bands were detected at approximately 50 kD and 25 kD (Fig 2A), consistent with previously published prenylated species recognised by anti-farnesyl antibodies [30] and (^3^H)prenyl/polyprenol labelling in malaria parasites [31,32]. The parasite prenylome includes prenylated proteins at the approximate sizes detected: Rab guanosine-5'-triphosphate (GTP)ases (23–27 kD) and a HSP40 analogue (PF3D7_1437900) (40 kD) [27,28]. Indolmycin-treated parasites, collected 72–78 hrs following drug treatment (equivalent to 28–34 hpi in the second IDC), had a significant reduction in Rab proteins detected by anti-farnesyl (*P* < 0.001, two-tailed Student *t* test) (Fig 2B), suggesting a reduced capacity for delayed-death parasites to prenylate target proteins in the IDC after treatment.

To determine if the apparent failure to prenylate protein in delayed-death parasites is due to lack of isoprenoids used for protein prenylation (i.e., FPP/GGPP), we substituted the polyprenol analogue geranylgeraniol (GGOH) into the medium of indolmycin-treated parasites. GGOH has previously been reported to protect parasites from fosmidomycin treatment [33]. We therefore hypothesised that GGOH substitution following indolmycin treatment would facilitate further protein prenylation by complementing the depletion of de novo prenyl groups. Consistent with this hypothesis, the indolmycin-treated parasites supplemented with 5 μM GGOH show bands at the same size as detected in the untreated sample (Fig 2A).

### Polyprenol substitution rescues parasites from delayed death

**Fig 3:**
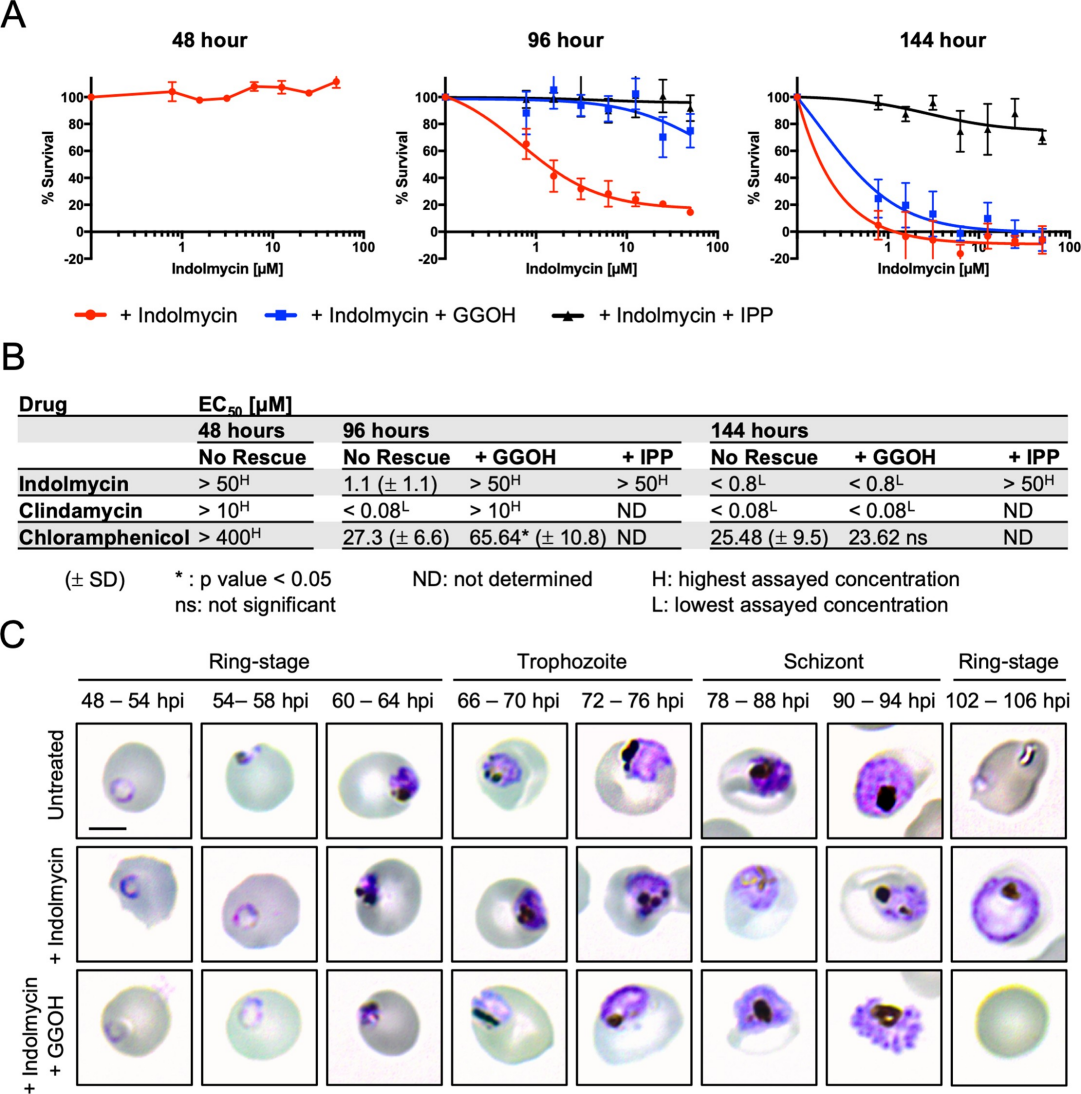
Polyprenol supplementation with GGOH protects parasites from the effect of apicoplast inhibitors in the second IDC after treatment. (A) Dose-response curve from SYBR-Green susceptibility assay determined 48, 96, and 144 hours post indolmycin treatment, with polyprenol (5 μM GGOH) or isoprenoid (200 μM IPP) supplementation as indicated. Indolmycin causes a delayed-death effect (inhibition at 96 but not 48 hours) that is rescued by GGOH or IPP supplementation. Inhibition at 144 hours is rescued by IPP but not GGOH supplementation. Data are presented as the means of three independent experiments ± SEM. See S2 Data for numerical data underlying figure. (B) Summary table of EC_50s_ for delayed-death antimalarials indolmycin, clindamycin, and chloramphenicol, determined at 48, 96, and 144 hours post treatment, with polyprenol (5 μM GGOH) or isoprenoid (200 μM IPP) rescue as indicated. Data are presented as the means of three independent experiments ± SD. *P < 0.05, two-tailed Student *t* test. (C) Thin-blood smears were collected for Giemsa-microscopy every 6 hours during the second IDC following indolmycin treatment. Scale bar = 5 μm. GGOH, geranylgeraniol; IDC, intraerythrocytic developmental cycle; IPP, isopentenyl pyrophosphate; ns, not significant.

Supplementing the medium of indolmycin-treated parasites with the isoprenoid precursor IPP (>200 μM) is sufficient to rescue parasites from delayed death indefinitely (Fig 3A) [18]. Growth inhibition of iRBCs by indolmycin has a 48-hour EC_50_ > 50 μM (*n* = 3), a 96-hour EC_50_ = 1.1 μM (*n* = 3, SD ± 1.1 μM), and a 144-hour EC_50_ = 0.1 μM (*n* = 3, SD ± 0.12 μM) (Fig 3B). With 200 μM IPP supplementation, iRBC are protected from the lethal effect of indolmycin up to the maximum concentration assayed (50 μM) at both 96 hours and 144 hours (Fig 3A and 3B). Given that GGOH supplementation protects against the depletion of prenylated proteins, we tested whether polyprenol precursors have a dose-dependent protective effect against indolmycin treatment. We first performed growth inhibition assays using GGOH and farnesol (FOH; the alcohol analogue of the prenyl group FPP) and determined that concentrations of GGOH exceeding 20 μM or FOH exceeding 30 μM were antagonistic to parasite growth (S1 Fig). To be used as productive substrates for protein prenylation, FOH and GGOH must first be phosphorylated (by unknown kinases) to FPP and GGPP; however, it is possible that FOH and GGOH compete directly as unproductive FPP and GGPP substrates and that this has a dose-dependent toxic effect on the parasite. Of these two polyprenol compounds, GGOH but not FOH showed protective effects when supplemented into the medium of indolmycin-treated parasites, with 5 μM GGOH being the lowest optimal protective concentration without antagonising parasite survival (S2A Fig). Likewise, supplementation of indolmycin-treated parasites with a combination of GGOH or FOH showed no synergistic protective effects (S2B Fig), suggesting that GGOH but not FOH is readily used as a prenylation substrate, which is consistent with ex vivo studies where [^3^H]GGOH was used preferentially to [^3^H]FOH as a substrate to modify protein in *P*. *falciparum* [32].

Supplementing the medium with 5 μM GGOH increased the 96-hour EC_50_ of indolmycin to >50 μM (*n* = 3) (Fig 3B). Importantly, GGOH was supplemented only after replacing the medium in the second IDC. However, unlike IPP, the protective effect of GGOH is only temporary, with no difference between the 144-hour EC_50_ values for indolmycin-treated and GGOH rescue (Fig 3B). This shows that provision of the polyprenol precursor GGOH rescues parasites during the second IDC following indolmycin treatment but that the rescued parasites still eventually succumb to the lethal effect of the drug precluding a third IDC (Fig 3C). We confirmed that temporary rescue with GGOH was common to delayed death–causing drugs by repeating the analysis with clindamycin and chloramphenicol, antibiotics that target separate components of the apicoplast protein-translation apparatus (Fig 3B), and which have previously been shown to be completely rescuable using IPP [34]. 5 μM GGOH supplementation protected parasites from delayed death up the maximum concentration of clindamycin assayed (>10 μM, *n* = 3, approximately 1,000× 96-hour EC_50_), and chloramphenicol showed a significant fold reduction in its 96-hour EC_50_ when treated parasites were supplemented with 5 μM GGOH from 27.3 μM, (*n* = 3, SD ± 6.6 μM) to 65.64 μM (*n* = 3, SD ± 10.8 μM) (*P* < 0.05, two-tailed Student *t* test) (Fig 3B and S3 Fig). However, consistent with our findings using indolmycin, GGOH did not protect parasites from treatment with clindamycin or chloramphenicol in the third IDC after treatment (Fig 3B and S3 Fig). Together, these data show that the failure to prenylate proteins during the parasite’s second IDC following indolmycin-treatment is the proximate cause of delayed death and that supplementing the polyprenol precursor GGOH is sufficient to alleviate the effects of isoprenoid fatigue in these parasites. However, restoring the capacity of delayed-death parasites to prenylate proteins alone is insufficient to indefinitely rescue parasites. As such, we hypothesise that disrupting protein prenylation is the immediate cause of parasite death in the second IDC but that even if this pathway is supplemented, another lethal insult ultimately arises, resulting in growth arrest that precludes a third IDC.

### Aberrant uptake/trafficking of RBC cytoplasm to the DV during delayed death

**Fig 4:**
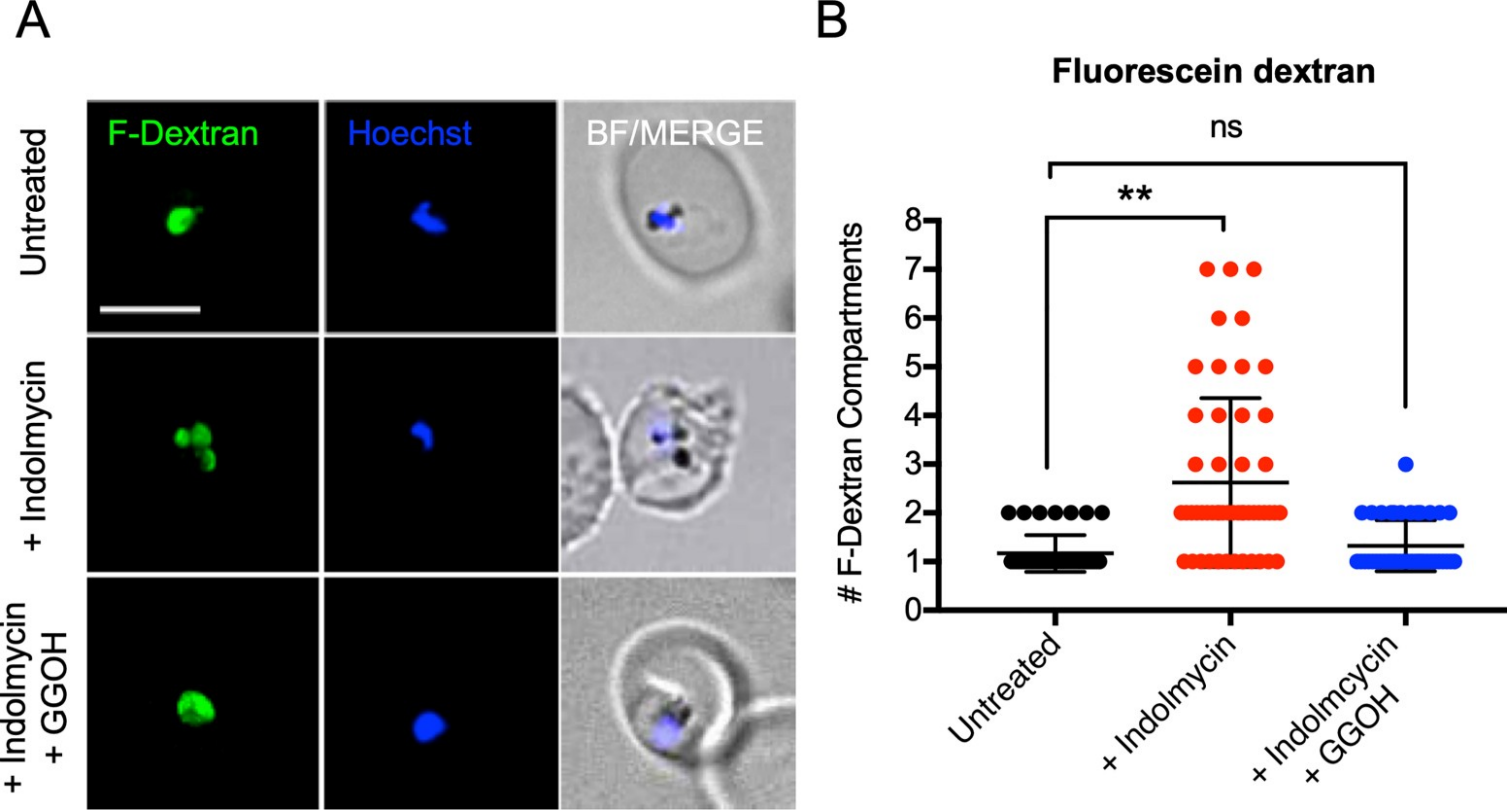
Indolmycin treated parasites aberrantly internalise F-dextran from preloaded RBCs in the second IDC after treatment. Uninfected RBCs were preloaded with F-dextran by gentle hypotonic lysis and resealing. Following this, enriched and synchronised schizont-stage parasites were added to the loaded RBCs and merozoites were allowed to reinvade. Newly invaded ring-stage parasites were treated with indolmycin (50 μM), with and without polyprenol (5 μM GGOH) rescue as indicated. (A) Representative live-cell images taken 72–78 hours after drug administration are shown (equivalent to 28–32 hpi in the second IDC after treatment). F-dextran, green signal; Hoechst: parasite nuclei, blue signal; merge: BF and blue signal. Scale bar = 5 μm. (B) The number of F-dextran compartments per iRBC were scored in three independent experiments. **P < 0.01, one-tailed Student *t* test. Scatter dot plot shows error bars with mean ± SD. See S2 Data for numerical data underlying figure. BF, bright field; F-dextran; fluorescein dextran; GGOH, geranylgeraniol; hpi, hours post invasion; IDC, intraerythrocytic developmental cycle; ns, not significant; RBC, red blood cell.

Considering the observed disruption in haemoglobin metabolism (Fig 1B), we sought to determine whether host haemoglobin uptake or trafficking is disrupted during delayed death. We investigated parasite-mediated RBC cytoplasm internalisation using a fluorescein-dextran (F-dextran) uptake assay modified from Frankland and colleagues [35] and Baker and colleagues [36]. Uninfected RBCs were loaded with F-dextran such that their cytosolic contents are fluorescently labelled. Newly invaded, preloaded iRBCs were treated with 50 μM indolmycin for 30 hours and imaged by live-cell fluorescence microscopy 72–78 hours following drug treatment (equivalent to 28–34 hpi in the second IDC) (Fig 4A). In the untreated iRBCs (DMSO only), the F-dextran was internalised by the parasite and concentrated into one or sometimes two compartments (Fig 4B), consistent with Baker and colleagues [36]. In parasites with two F-dextran-labelled compartments, the larger compartment was typically adjacent to a smaller compartment (Fig 4A, untreated). This is consistent with a single parasite DV and transitory vesicles containing F-dextran internalised from the RBC cytoplasm. In the indolmycin-treated group, the F-dextran was more frequently concentrated into multiple compartments (*P* < 0.01, one-tailed Student *t* test) (Fig 4B). This suggests that although the initial internalisation of RBC cytoplasm by treated parasites is unaffected, the trafficking or fusion of the internalised vesicles to the DV is disrupted. This led to an accumulation of aberrant compartments that is consistent with a defective RBC cytoplasm uptake phenotype [37]. Parasites treated with indolmycin but then supplemented with GGOH after treatment internalised the F-dextran into one or two compartments, equivalent to untreated samples (Fig 4B). This demonstrates that delayed death is linked to the aberrant uptake of RBC cytoplasm, but this is ameliorated by polyprenol substitution, which suggests that the vesicle trafficking defect is prenylation dependent.

### Ultrastructural analysis of the parasite DV reveals fragmented biogenesis

**Fig 5:**
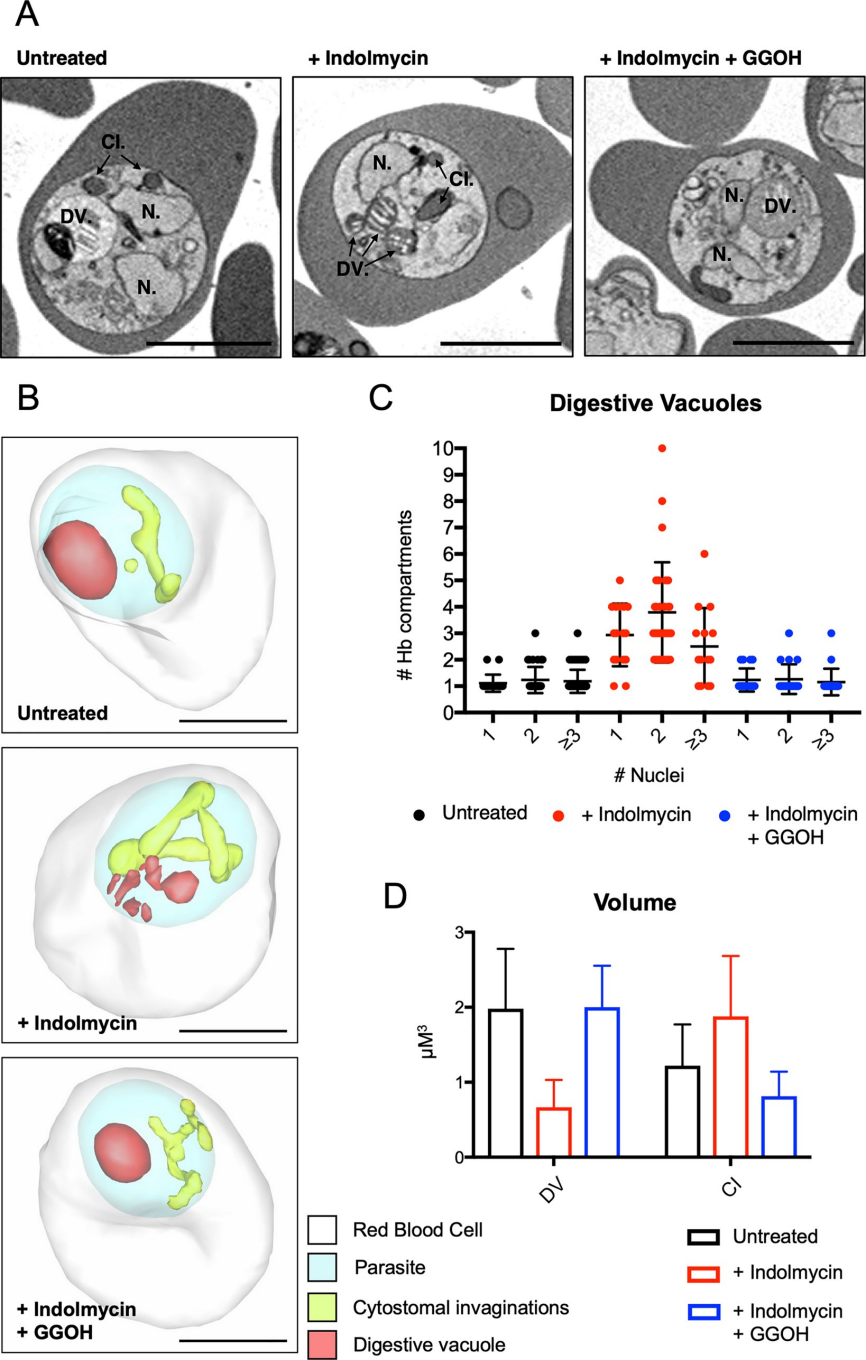
Aberrant morphology of intracellular structures is prenylation dependent in delayed-death parasites. Parasites were treated with indolmycin (50 μM), with and without polyprenol rescue (5 μM GGOH) as indicated. Enriched trophozoite-stage parasites were collected for reduced osmium fixation 72–78 hours post drug administration (equivalent to 28–32 hpi in the second IDC after treatment). (A) Representative images (top-down and cross-sectional), from SEM of each condition: untreated, indolmycin treated (+ indolmycin), and indolmycin treated with polyprenol rescue (+ indolmycin + GGOH). Structures indicated are N, DV, and CI. Scale bar = 3 μm. (B) 3D-rendered iRBCs using serial block-face scanning electron microscopy. Indicated compartments are RBC (white), parasite (blue), CIs (yellow), and DV (red). Scale bar = 3 μm. (C) Parasites were scored for number of DV compartments relative to number of nuclei in each treatment condition. Untreated: 1 nucleus *n* = 18, 2 nuclei *n* = 34, ≥ 3 nuclei *n* = 49; + indolmycin: 1 nucleus *n* = 16, 2 nuclei *n* = 29, ≥ 3 nuclei *n* = 14; + indolmycin + GGOH 1 nucleus *n* = 17, 2 nuclei *n* = 19, ≥ 3 nuclei *n* = 19. Scatter dot blots show error bars with mean ± SD. (D) The total volume (μm^3^) of the DVs and CIs were measured in each treatment condition. Bar graphs show error bars with mean ± SD. See S2 Data for numerical data underlying figure. CI, cytostomal invagination; DV, digestive vacuole; GGOH, geranylgeraniol; hpi, hours post invasion; IDC, intraerythrocytic developmental cycle; N, nucleus; RBC, red blood cell.

Internalised RBC cytoplasm accumulates within multiple aberrant compartments of the delayed-death parasites. To investigate the nature of these aberrant compartments, we performed an analysis of parasite ultrastructure by electron microscopy. Initial analysis of ultrathin sections of indolmycin-treated parasites by transmission electron microscopy revealed aberrant DVs as well as multiple membrane-bound enclosures of RBC cytoplasm (S4 Fig). The 3D arrangement of such aberrant compartments is difficult to decipher from individual sections, so we examined the 3D ultrastructure of untreated and delayed-death parasites using reconstruction of hundreds of sections after serial block-face scanning electron microscopy (SEM) (Fig 5 and S1 Movie). 3D reconstructions of multiple trophozoite-infected RBCs supported our initial observations from 2D electron microscopy sections (Fig 5A)—that indolmycin treatment disrupts formation of the DV and that treated parasites generate multiple DV fragments, each containing haemozoin crystals (Fig 5B and S2 Movie). Remarkably, GGOH rescue completely ameliorated the fragmented DV phenotype, restoring a single DV (Fig 5B). Quantification of the number of DV compartments per cell in different conditions is shown in Fig 5C. This suggests a hereto unknown role for prenylated proteins in the formation of a mature DV.

As well as confirming the fragmentation of the DV in delayed-death parasites, analysis of the 3D reconstructions dramatically altered our understanding of the membrane-bound enclosures of iRBC cytoplasm in the delayed death parasites. Rather than representing numerous discrete compartments, these were revealed as dramatic extensions of the cytostomal invagination that twisted and wound throughout the cytoplasm of the parasite (Fig 5B, and S3–S5 Movies). As with the DV fragmentation phenotype, this extended invagination phenomenon was rescued by addition of GGOH. We found that the total volume of DVs decreased while the volume of the cytostomal invaginations increased within the indolmycin-treated parasites compared to untreated and GGOH-rescued parasites (Fig 5D). These data support a model whereby the trafficking of RBC cytoplasm (and thus haemoglobin) from cytostomes is perturbed such that the cytostomal invagination itself becomes distended, and the delivery of packages of RBC cytoplasm is severely impaired resulting in a fragmented DV.

### Rab GTPase-mediated vesicular trafficking is disrupted during the second IDC following indolmycin treatment

As demonstrated above, delayed death diminishes protein prenylation during the second IDC following treatment (Fig 2). We hypothesised that without prenyl modification of nascent protein, the protein substrates of prenyl-transferases that are dependent on their prenyl modification for localisation or function, such as Rab GTPases, will mislocalise during delayed death. We examined the prenylated Rab GTPase, Rab5a, which has previously been shown to be associated with haemoglobin-containing vesicles and has been hypothesised to play a role in at least one of the three or four semiredundant, but unique haemoglobin uptake pathways present in *P*. *falciparum* [29]. More recent experiments in *Toxoplasma* indicate a role for Rab5a in the vesicular trafficking to the secretory organelles [38]. It remains unclear what role Rab5a has in *Plasmodium* vesicular trafficking; nevertheless, it is a useful representative vesicular trafficking determinant whose function relies on prenylation [39].

**Fig 6:**
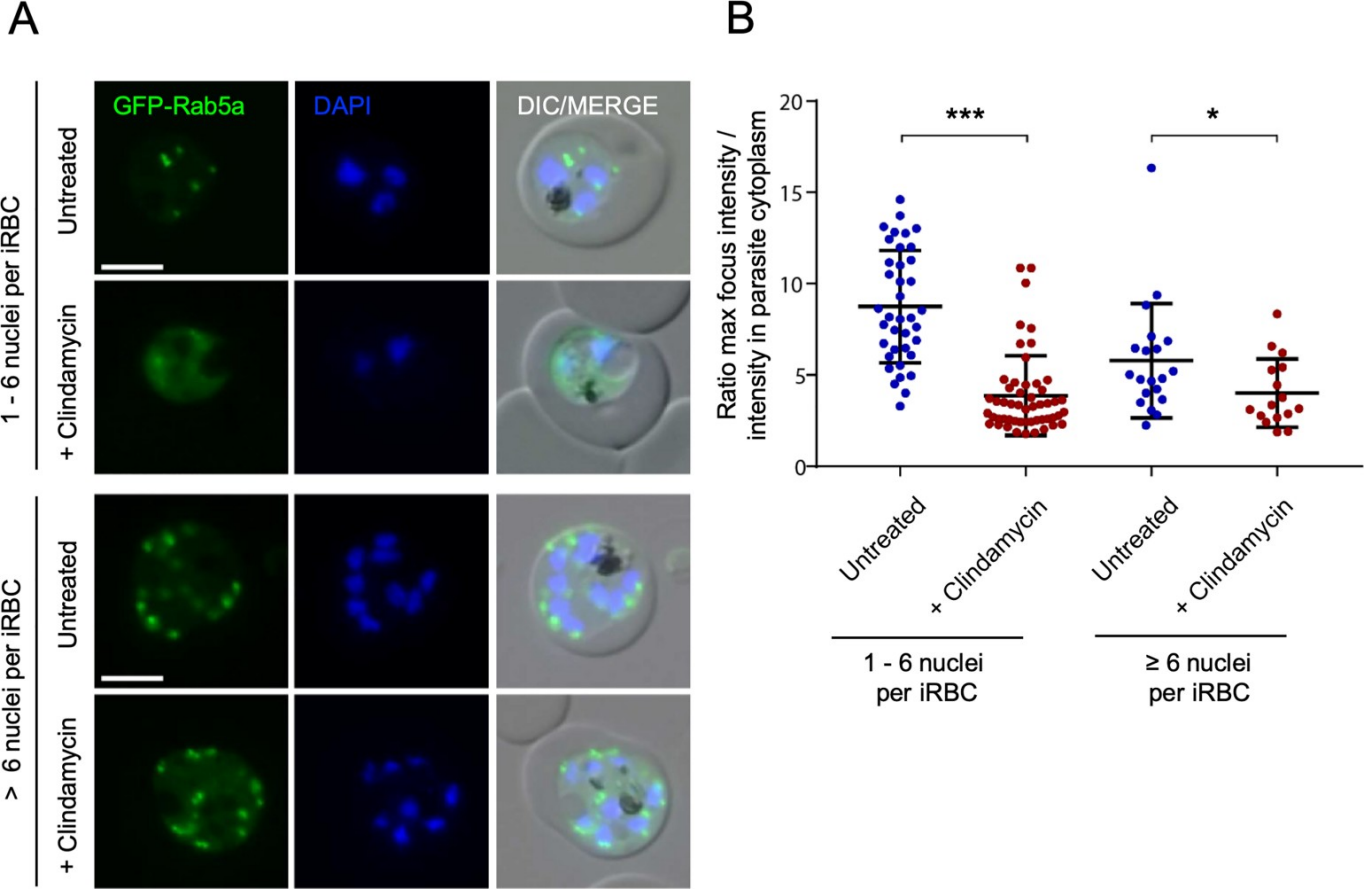
Rab5a shows an aberrant localisation in the second IDC after clindamycin treatment. (A) Representative live-cell images of untreated and clindamycin (5 μM) treated parasites in the second IDC following treatment are shown. Cell populations were divided into parasites with 6 or fewer nuclei (upper panel) and parasite with more than 6 nuclei (lower panel), as the disruption is clearly most pronounced in trophozoite stage parasites. GFP-Rab5a, green signal; DAPI: parasite nuclei, blue signal; merge of green and blue signal. Scale bar = 5 μm. (B) Graph showing the ratio of the brightest GFP focus fluorescence intensity to mean fluorescence intensity distributed in the parasite cytoplasm in untreated and clindamycin treated parasites. *N* = 38 for untreated, 1–6 nuclei; *n* = 52 for + clindamycin, 1–6 nuclei; *n* = 20 for control, > 6 nuclei and *n* = 16 for + clindamycin, > 6 nuclei. *P < 0.05; ***P < 0.001, Welch *t* test. Scatter dot plots show error bars with mean ± SD. See S2 Data for numerical data underlying figure. DAPI, 4′,6-diamidino-2-phenylindole; DIC, differential interference contrast; GFP, green fluorescent protein; IDC, intraerythrocytic developmental cycle; iRBC, infected red blood cell.

We detected green fluorescent protein (GFP)-fusions of Rab5a in untreated parasites as discrete foci dispersed throughout trophozoite-stage parasites (Fig 6), as previously shown [39]. During the second IDC following clindamycin treatment, GFP-Rab5a mislocalised and, instead of being concentrated in discrete foci, was dispersed in a diffuse cytoplasmic pattern. Prenylation of Rab5a mediates membrane association (possibly alongside cargo-binding), and the apparent cytosolic dispersal of Rab5a in delayed-death parasites is consistent with loss of prenylation-mediated membrane attachment. This effect was less pronounced in late-stage parasites (Fig 6), suggesting that Rab5a’s localisation may be influenced by additional factors in that stage.

**Fig 7:**
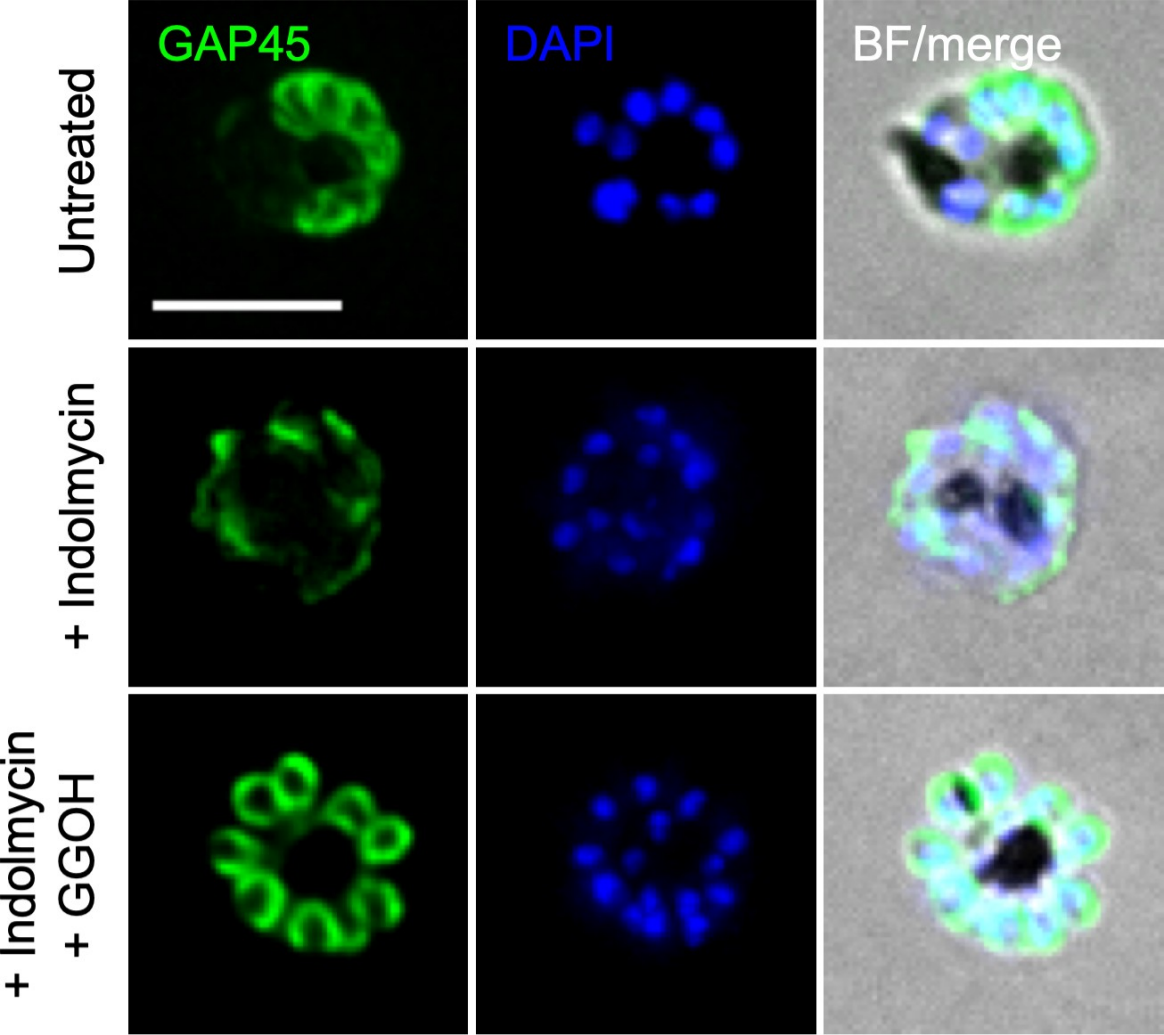
IMC formation is disrupted in the second IDC after indolmycin treatment. Shown are representative images of immunofluorescence assays using anti-GAP45 (1:1,000). Untreated, indolmycin (50 μM) treated and indolmycin treated with polyprenol rescue (5 μM GGOH) as indicated, collected in the second IDC after treatment. The IMC marker GAP45 localises atypically in indolmycin treated parasites. GGOH supplementation restores GAP45 localisation to the IMC equivalent to untreated. GAP45, green signal; DAPI, parasite nuclei, blue signal; merge of green and blue signal. Scale bar = 5 μm. BF, bright field; DAPI, 4′,6-diamidino-2-phenylindole; GGOH, geranylgeraniol; IDC, intraerythrocytic developmental cycle; IMC, inner membrane complex.

Another prenylated Rab, Rab11a, is localised to the inner membrane complex (IMC) of *P*. *falciparum* and is proposed to facilitate vesicle recycling and transport of IMC proteins such as glideosome-associated protein 45 (GAP45) [40]. Consistent with previous observations [41], we observed GAP45 at the IMC in schizont stages of untreated parasites. However, following indolmycin treatment during the second IDC, GAP45 did not localise to structures that resemble IMC (Fig 7), suggesting that there is a defect in the assembly or trafficking of the IMC in delayed death parasites. The normal localisation of GAP45 was restored with GGOH recue treatment (Fig 7). Importantly, secretory trafficking pathways remain intact in the second IDC following indolmycin or clindamycin treatment, as demonstrated by normal export of ring-exported protein 1 (REX1) to the iRBC (S5 Fig). The prenylation-dependent mislocalisation of Rab5a to the cytosol and putative disruption of Rab11a cargo delivery suggests that these trafficking proteins lose their normal association with target biological membranes during delayed death, consistent with the inactive or unbound form of these Rab GTPase proteins.

### Indolmycin-induced delayed death increases osmotic fragility of iRBC

Because indolmycin treatment produces severe disruption of the morphology of the DV and haemoglobin trafficking apparatuses, we examined potential physiological effects from disrupted feeding that may contribute to delayed death. Intraerythrocytic parasites progressively internalise and digest host-derived haemoglobin to 1) acquire amino acids [42], 2) increase the available space within the iRBC into which the parasite can grow into [43], and 3) catabolise and expel excess host protein to mitigate the increased colloid-osmotic pressure caused by parasite growth within the iRBC [44,45]. Aberrant haemoglobin uptake induced by indolmycin treatment might kill parasites by disrupting these homeostatic mechanisms.

**Fig 8:**
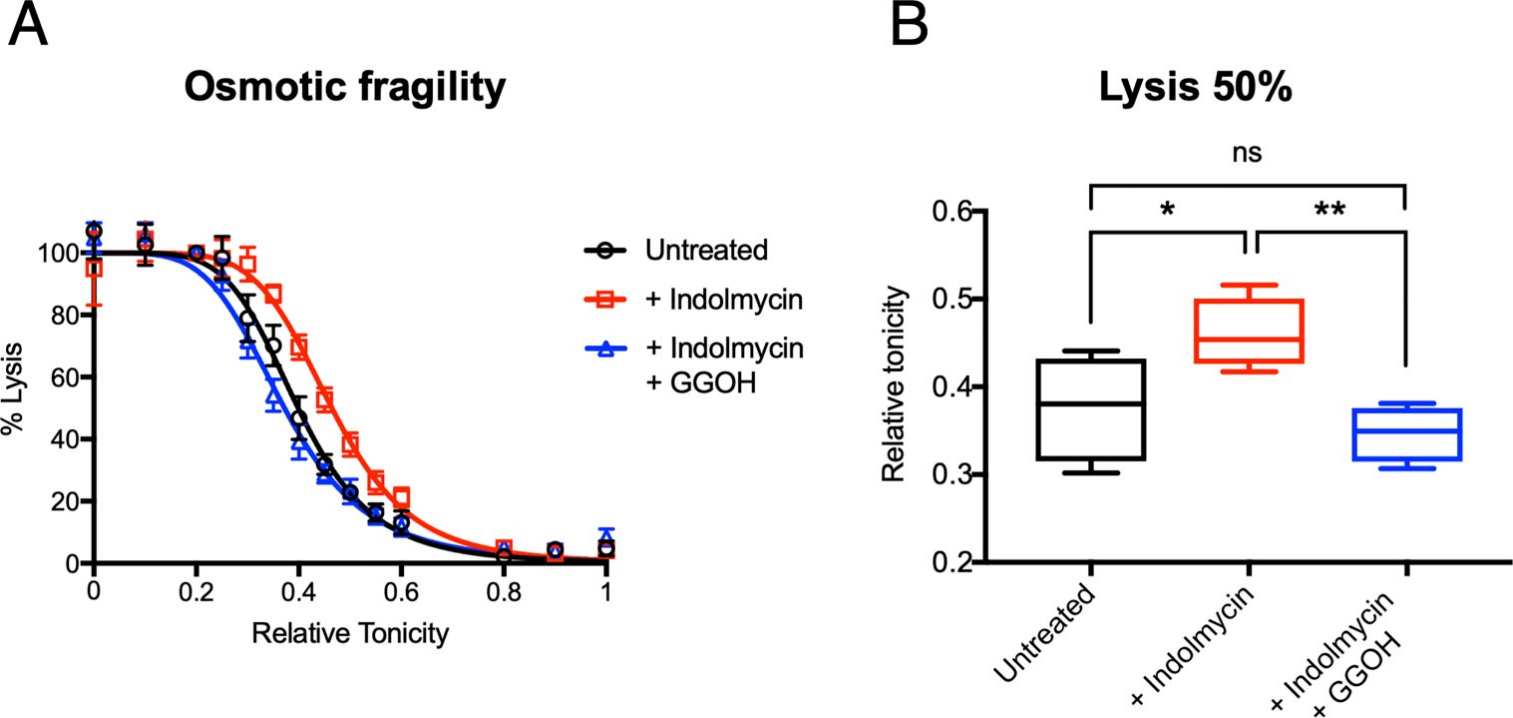
Indolmycin-treated parasites have increased osmotic fragility in the second IDC after treatment. (A) Parasites were treated with indolmycin (50 μM) and divided into two conditions, with and without polyprenol rescue (5 μM GGOH). Enriched trophozoite-stage parasites were collected 72–78 hours post drug administration (equivalent to 28–32 hpi in the second IDC after treatment) and incubated in solutions with tonicity varied by increasing the concentration of NaCl. Percentage iRBC lysis was calculated by measuring the absorbance at 415 nm (A_415_) of released haem normalised to a relative tonicity of one (isotonic). Representative of four independent experiments. Scatter plots show error bars with mean ± SEM. See S2 Data for numerical data underlying figure. (B) The concentration required for Lysis C_50%_ for each of the three conditions: untreated, indolmycin treated (+ indolmycin), and polyprenol rescue (+ indolmycin + GGOH). The Lysis C_50%_ for indolmycin treated parasites increased by 22.3% (*n* = 4, SD ± 6.07) whereas the Lysis C_50%_ for the polyprenol rescued parasites did not significantly change. *P < 0.05; **P < 0.01; two-tailed Student *t* test. Box and whisker plots show the interquartile range. See S2 Data for numerical data underlying figure. GGOH, geranylgeraniol; hpi, hours post invasion; IDC, intraerythrocytic developmental cycle; Lysis C_50%_, half-maximal lysis; ns, not significant; RBC, red blood cell.

To determine whether the cellular homeostasis that maintains osmotic pressure is disrupted in indolmycin-treated parasites, we measured osmotic fragility. The propensity for iRBCs to haemolyse in varying hypotonic solutions was measured using a modified method described in Mauritz and colleagues [45] and Dennis and colleagues [46]. Indolmycin-treated parasites showed an increase in osmotic fragility relative to untreated iRBC at all tonicities assayed in the second IDC after treatment (Fig 8A). The half-maximal lytic concentration for indolmycin treated parasites increased significantly by 22.3% (*n* = 4, SD ± 6.07) (*P* < 0.05, two-tailed Student *t* test) (Fig 8B). Like other experiments described above, restoration of prenylation by rescue with GGOH restored measurements of osmotic fragility to those of untreated iRBCs (Fig 8A and 8B). This supports the suggestion that disruption of protein prenylation by indolmycin treatment reduces the capacity of treated parasites to internalise and degrade host-derived haemoglobin and that this has a profound effect on the physiology of the parasite and its host cell, likely contributing to growth arrest and delayed death.

### Uncoupling ubiquinone and dolichol contributions during third IDC arrest

Restoring protein prenylation in delayed death using GGOH rescue treatment demonstrates that the proximate cause of delayed death is a defect in protein prenylation. However, even when rescued by GGOH, these polyprenol-rescued parasites are still unable to survive a third IDC following treatment (Fig 2 and S3 Fig) suggesting that additional causes of parasite death arise from disruption of another isoprenoid pathway. Ubiquinone (or Co-enzyme Q) is the principle and essential electron acceptor for electron transport in mitochondrion. Ubiquinone inserts into the inner membrane of the mitochondrion by its isoprene tail. In blood-stage *P*. *falciparum*, the mitochondrion’s primary function is to provide electrons for use by dihydroorotate dehydrogenase, an essential metabolic enzyme required for pyrimidine biosynthesis [47]. To determine whether disruption of ubiquinone contributes to third IDC arrest in polyprenol-rescued parasites, we used a transgenic parasite expressing *Saccharomyces cerevisiae* (yeast) dihydroorotate dehydrogenase (yDHODH). The yDHODH enzyme uses fumarate as an electron acceptor and is independent of ubiquinone for pyrimidine biosynthesis and hence uncouples blood-stage *P*. *falciparum*’s dependence on mitochondrial electron transport [47]. Although these transgenic yDHODH parasites are slow growing, in asexual blood stages, they are resistant to inhibition that results in mitochondrial perturbations.

**Fig 9:**
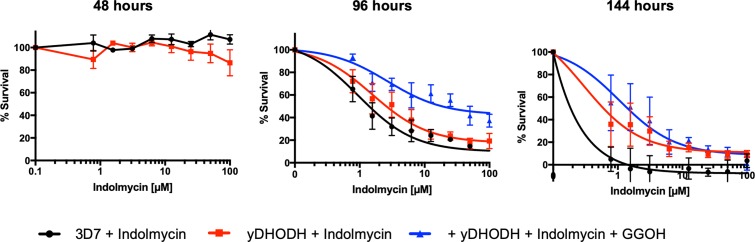
Supplementing ubiquinone-independent yDHODH-expressing transgenic parasites with GGOH does not protect from delayed death in the third IDC after treatment. Dose-response curve from SYBR-Green susceptibility assay determined 48, 96, and 144 hours post indolmycin treatment, with and without polyprenol (5 μM GGOH) rescue. Assays were performed in parallel with 3D7 and transgenic parasites expressing ubiquinone-independent yDHODH. yDHODH parasites are susceptible to delayed death equivalent to 3D7 (inhibition at 96 hours). GGOH supplementation does not further protect yDHODH parasites in the third IDC (inhibition at 144 hours). Representative of at least three independent experiments. Scatter plots show error bars with mean ± SEM. See S2 Data for numerical data underlying figure. GGOH, geranylgeraniol; IDC, intraerythrocytic developmental cycle; yDHODH, yeast dihydroorotate dehydrogenase.

Parasites expressing yDHODH were not innately protected from second IDC arrest after indolmycin treatment (Fig 9). This suggests that isoprenoid disruption of parasite DHODH is not the cause of delayed death and indeed causes no further insult in the second IDC after indolmycin treatment even after the initial insult of disruption of protein prenylation. Furthermore, indolmycin-treated yDHODH parasites supplemented with GGOH were not protected in the third IDC after treatment. This result indicates that arrest in the third IDC after treatment cannot be attributed either to disruption of parasite DHODH or protein prenylation. The remaining known fate for isoprenoids in *Plasmodium* is in the synthesis of dolichols, so *P*. *falciparum* survival during the third IDC following indolmycin treatment is likely co-contingent with dolichol biosynthesis from isoprenoid precursors.

## Discussion

Apicoplast-located isoprenoid precursor biosynthesis is essential for parasite survival, but the downstream effects on parasite cellular processes brought about by loss of apicoplast function have not been elucidated. We hypothesised that second IDC arrest in delayed-death parasites arises from a defect in the apicoplast that ablates IPP biosynthesis, which lethally effects parasite cellular processes by depressing the turnover of essential isoprenoid products: prenyl groups, ubiquinone, or dolichol. We refer to the cessation of de novo isoprenoid biosynthesis and exhaustion of available products as an isoprenoid fatigue. Furthermore, we aimed to dissect the temporal contribution of disrupting each of these pathways in delayed death to define the hierarchy of causes for parasite growth arrest. Here, we argue that the proximate cause of delayed death is the depletion of prenyl substrates required for protein prenylation, precipitating functional disruptions to intracellular trafficking, IMC assembly, DV biogenesis, and perturbing cellular homeostasis.

Disruption of prenylation-dependent processes generates parasite abnormalities that are apparent by the trophozoite stage, with diminished haemoglobin turnover and aberrant DV morphology. However, treated parasites branch their mitochondrion as usual during the trophozoite stage [48] and transition to early schizont stages, completing multiple rounds of nuclear division (e.g., Figs 6 and 7). This, combined with the finding from the metabolomic analyses of gradual depletion of isoprenoids during the second cycle, suggests that the effect of ablating protein prenylation by isoprenoid depletion is cumulative rather than immediate. This interpretation is consistent with the findings by Chakrabarti and colleagues [32], who showed through labelling experiments that most prenlyation occurs during the trophozoite to schizont transition, and Nallan and colleagues [49], who showed that prenyl-transferase inhibitors arrest parasites at the trophozoite stage. Our data suggest that delayed death overlaps with but is distinct from phenotypes induced by fosmidomycin-mediated inhibition of IPP biosynthesis during the terminal cycle [30]. Fosmidomycin is an inhibitor of the MEP/DOXP pathway [33], and fosmidomycin treatment leads to a sudden arrest in IPP biosynthesis and parasite death at a similar life stage, albeit in the first IDC with treatment, coincident with disruption of prenylation [30]. If fosmidomycin were added early enough in the cycle, we anticipate that it would also produce comparable effects on haemoglobin turnover DV morphology in the same cycle. In contrast, delayed-death drugs have no noticeable effect on the abundance or function of the apicoplast MEP/DOXP biosynthetic enzymes. Instead, we propose that delayed-death drugs lead to a gradual expenditure of residual IPP in the second IDC—following the loss of the apicoplast—that eventually lead to the loss of prenylation.

A pair of recent labelling experiments reveals a limited number of prenylated proteins that are likely defective in delayed-death parasites. Fewer than 20 prenylated proteins have been identified, predominantly Rab GTPases, with canonical and putative roles in vesicular membrane trafficking [27,28]. The restricted prenylome of *P*. *falciparum* compared to other higher eukaryotes indicates that prenylated proteins have relatively few roles in regulating parasite cellular processes. These restricted roles are consistent with our observation of only limited morphological and metabolic aberrations in parasites unable to synthesis new prenyl groups.

The temporary rescue of the prenyl-dependent delayed defects by GGOH (Figs 2, 4, 5 and 8) confirms the metabolomic finding that apicoplast-generated IPP is used for higher isoprenoid compounds, and their depletion is the proximate cause of delayed death. The short-chain length prenyl groups FPP and GGPP are the substrates for prenyl-transferases that covalently modify protein by attaching prenyl moieties to cysteine residues at the carboxyl terminus of target proteins. The successful rescue from delayed death by the polyprenol analogue GGOH but not FOH is consistent with delayed death being dependent on prenylation of the *Plasmodium*-Rab GTPases, which possess C-terminal prenylation motifs that predict attachment of geranylgeranyl rather than farnesyl moieties, which were identified as possessing GGPP tags in the *Plasmodium* prenylomes [27,28].

Prenylated Rab GTPases are master coordinators of intracellular vesicular trafficking. Rabs act as molecular switches to regulate membrane rearrangements and facilitate vesicle budding, motility, and fusion by recruiting effector proteins in their GTP-bound state [50]. The prenyl modifications of Rab proteins are predicted to mediate their anchoring to the lipid bilayer of endomembranes. Chemical or genetic ablation of the prenyl modification on Rab5a or FYVE-containing coiled–coil protein (FCP; localised to the DV) leads to the mislocalisation of these proteins [28,30,39], demonstrating that prenylation is necessary for correct protein function. We found that Rab5a mislocalised during delayed death and that this coincided with a haemoglobin trafficking defect. Previous conditional inactivation of Rab5a alone did not recapitulate this phenotype [29,39], suggesting that the disruption of multiple prenylated proteins in the delayed-death parasites are responsible for the perturbation of cytostomes and DV biogenesis. Consistent with our observation, blockade of isoprenoid biosynthesis by fosmidomycin specifically disrupts the ultrastructure of the DV [30]. Our study suggests that multiple proteins in *P*. *falciparum*, prenylated using apicoplast isoprenoids, are essential for normal DV development and by extension metabolism of host haemoglobin.

We also found that IMC assembly, previously shown to require vesicular trafficking by the prenylated Rab11a, was disrupted during early schizogony. The mature IMC marker GAP45 has been colocalised to Rab11a-positive vesicles, indicating a role for Rab11a in trafficking IMC proteins [40]. Hence, we hypothesise that mislocalisation of Rab11a in delayed death could directly inhibit IMC assembly. Indeed GAP45, which associates with membranes via N-terminal myristylation and palmitoylation rather than prenylation, was also mislocalised in treated parasites, suggesting that overall assembly of the IMC is compromised in delayed death. This is supported by the failure of delayed-death parasites to segment into merozoites during second IDC arrest, as confirmed by light microscopy (Fig 2C).

Haemoglobin degradation is integral to parasite homeostasis, and disequilibrium of this metabolic pathway likely contributes to delayed death in *P*. *falciparum*. The parasite faces two physiological challenges as it grows within an iRBC: obtaining sufficient space and maintaining osmotic pressure. In human RBCs, haemoglobin comprises 95% of the total protein in the cytoplasm [43]. As the parasite grows, its volume increases, but the average total volume of the enclosing iRBC remains largely unchanged [43,51,52]. To achieve this, the parasite must internalise RBC cytoplasm by endocytosis and reduce the RBC contents. One reason for mitigating total iRBC volume expansion could be to prevent the volume of an iRBC increasing such that it surpasses its lytic volume and prematurely haemolyses (or is cleared by the spleen).

Aside from spatial restrictions, the uptake and turnover of host haemoglobin is thought to play an important role in mitigating increasing osmotic stress on an iRBC as the parasite grows [44,45]. The metabolically active trophozoite stage of *P*. *falciparum* massively increases the permeability of the iRBC membrane, inducing the expression of the new permeation pathways (NPPs) [53]. However, the net osmotic gain of this change should be sufficient to cause the iRBC to haemolyse [54]. The parasite must therefore compensate by reducing the osmotic influx of water by decreasing the colloid–osmotic pressure exerted by proteins within the cell [45]. In effect, the parasite ingests and proteolyses host haemoglobin to amino acids that it can release across the iRBC membrane via the NPPs [55]. We therefore hypothesise that disruption of haemoglobin internalisation and metabolism in delayed-death parasites must increase the osmotic fragility of the iRBC. Consistent with this, iRBCs treated with indolmycin did haemolyse more readily in hypotonic solutions (Fig 8), suggesting that prenylation-dependent trafficking defects do perturb osmolyte homeostasis. This may be even more pronounced during the turbulence experienced during an in vivo infection, but such haemolysis is difficult to measure. Our results would suggest that liver-stage–derived merozoites with defective apicoplasts will also lack appropriate prenylation and likely succumb in a similar manner to blood-stage delayed-death parasites.

The delayed-death effect observed in *Plasmodium* also has implications for understanding the similar phenotype in *Toxoplasma*. As in *Plasmodium*, *Toxoplasma* relies on the apicoplast for isoprenoid synthesis, albeit with additional isoprenoid scavenging from the host cell [19]. We anticipate that prenyl-dependent defects brought about by apicoplast delayed death also impact endocytosis in *Toxoplasma* and likely disrupt feeding. However, there is not a direct 1:1 orthologue match for all pairs of *Plasmodium* and *Toxoplasma* Rabs [38], and the digestion of haemoglobin in the *Plasmodium* vacuole in particular has no obvious direct cognate in *Toxoplasma*. Unlike *Plasmodium* blood stages, *Toxoplasma* tachyzoites are also dependent on their apicoplast for fatty acid biosynthesis, and this probably creates additional differences in the mechanism of *Toxoplasma* delayed death.

Disrupting isoprenoid biosynthesis perturbs several downstream cellular processes, and we sought to dissect how perturbation to each pathway contributes to delayed death. Disruption of protein prenylation represents the proximate cause of delayed death during the second IDC after treatment with a plastid inhibitor. However, arrest in the third IDC following treatment implies that isoprenoid fatigue also lethally perturbs other isoprenoid pathways in *P*. *falciparum*, i.e., synthesis of the ubiquinone isoprenoid-sidechain and dolichols. Using GGOH-rescued parasites, we uncoupled the essential role of protein prenylation from the contribution of isoprenoid products ubiquinone or dolichol. We determined that the yDHODH parasite line remained sensitive to second IDC arrest following indolmycin treatment and that cotreatment with GGOH was insufficient to protect parasites further. IPP provided by the apicoplast would normally be incorporated into dolichols required for GPI anchor biosynthesis, but this pathway is not rescued by GGOH. The essential contribution of dolichols and thus GPI-anchored proteins to establishment of a third IDC likely therefore explains the inability of GGOH to provide a longer-lasting rescue.

**Fig 10:**
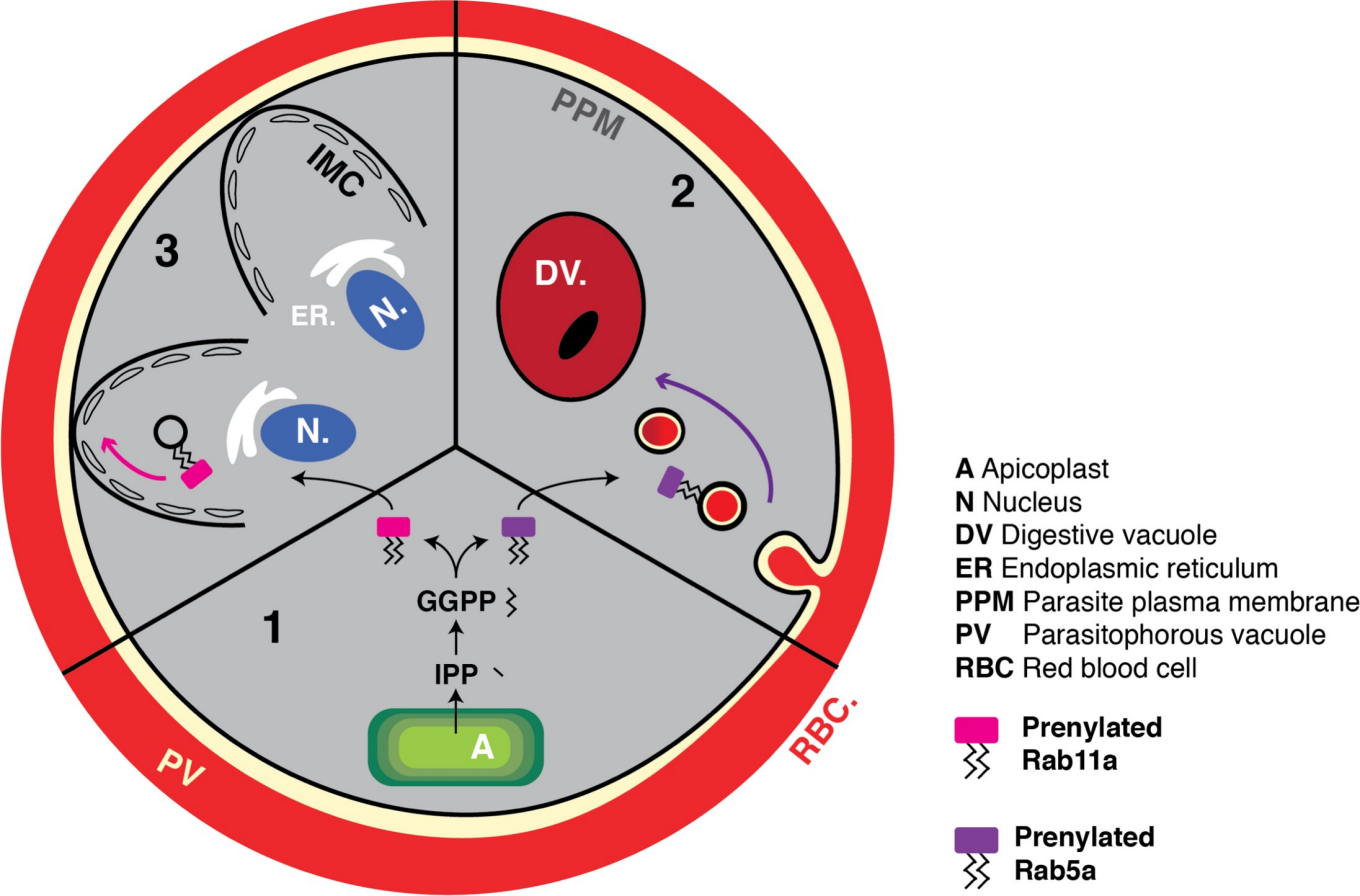
Multifaceted disruption of protein prenylation by isoprenoid fatigue during delayed death. (1) IPP is synthesised by the apicoplast to produce the prenyl-group GGPP. Protein substrates are modified by prenylation through the covalent attachment of one or more prenyl groups to their C-terminal cysteine residues. Prenylated proteins in *P*. *falciparum* have canonical roles in endomembrane vesicle trafficking. (2) The early endosome marker Rab5a is a prenylated protein in *P*. *falciparum* putatively involved in haemoglobin-containing vesicle trafficking to the parasite DV. Depletion of IPP during delayed death disrupts protein prenylation and Rab5a mediated vesicle trafficking to the DV. (3) Prenylated Rab11a facilitates the assembly of the IMC by trafficking cargo, including the IMC protein GAP45 to the IMC during merozoites segmentation. Depletion of IPP during delayed death disrupts the formation of the IMC. DV, digestive vacuole; GAP45, glideosome-associated protein 45; GGPP, geranylgeranyl pyrophosphate; IMC, inner membrane complex; IPP, isopentyl pyrophosphate.

Taken together, we can now establish a timeline for cellular processes subject to isoprenoid fatigue that leads to parasite arrest in delayed death (Fig 10). The indolmycin-treated parasite completes its first IDC normally but transmits a defective apicoplast to its progeny. This apicoplast may be capable of producing some isoprenoid precursor at the start of the second IDC, but this capacity wanes over the early stages of the cycle, and the isoprenoid products necessary for generating prenyl groups become depleted. Without available prenyl groups, nascent trafficking proteins are no longer modified by prenylation and mislocalise, losing their functionality. Haemoglobin trafficking, inner membrane complex formation, and merozoite membrane biogenesis all require effective protein prenylation; during delayed death, these processes are perturbed, which leads to second IDC parasite arrest. If parasites are treated with the polyprenol substrate GGOH, protein prenylation is recovered and parasites survive and segment. However, even in the presence of GGOH, parasites are unable to progress through a complete third IDC, likely due to disruption of dolichol mediated GPI synthesis and/or ubiquinone electron transport, which are not chemically rescued by GGOH.

## Methods

### *P*. *falciparum* culture

*P*. *falciparum* 3D7 parasites were maintained in continuous culture with minor modifications to the method described by [56]. Briefly, parasites were cultured in human O+ RBCs (leukocyte-depleted by filtration, provided by the Australian Red Cross Blood Service) at 2% haematocrit in RMPI-1640 supplemented with 25 mM sodium bicarbonate, 25 mM HEPES, 150 μM hypoxanthine, 20 μg/mL gentamicin, and 0.5% (w/v) Albumax II (Invitrogen) (complete medium). Cultures were maintained in flasks and petri dishes inside a sealed container filled with a low oxygen malaria-mix gas (1% O_2_, 5% CO_2_, and 94% N_2_) at 37°C with or without mechanical shaking.

Synchronised ring-stage cultures were obtained by double (14-hr interval) treatment with 5% (w/v) D-sorbitol during the parasite’s IDC prior to experimentation, and a single sorbitol treatment of ring-stage parasites immediately prior to experimentation. Alternatively, 0–4 hpi ring-stage parasites were generated by first enriching for schizonts using a custom magnetic separation apparatus described previously [57], incubating the enriched parasites with fresh RBCs for 4 hrs, and then treating the newly invaded RBCs with 5% (w/v) D-sorbitol.

### LC-MS metabolic profiling

Metabolite profiling across the development of delayed death was initiated by treating 0–4-hr ring-stage cultures (prepared as described above) with 50 μM indolmycin (BioAustralis Fine Chemicals Product 21200-24-8) or dimethyl sulfoxide vehicle control for 30 hrs and then the culture medium was replaced. Samples were collected at 30, 58, 68, 78, and 88 hrs post drug administration (equivalent to 30 hpi in the first IDC and then 14, 24, 34, and 44 hpi in the second IDC) for metabolite extraction and analysis by LC-MS. Samples with equivalent parasitemia were collected and 1×10^8^ RBCs per conditions were centrifuged at 14,000 g for 30 seconds (4°C), washed with 1 mL of ice-cold phosphate buffered saline (pH 7.4), centrifuged again and the cell pellet extracted with 200 μL of 80% (v/v) acetonitrile (in water containing 5 μM ^13^C-aspartate as the internal standard). The cell extracts were rapidly resuspended, vortexed and then centrifuged at 14,000 g for 10 min at 4°C. The metabolite extract was transferred to an MS vial and LC-MS analysis was preformed using methods previously described [58], with an Agilent Q-TOF mass spectrometer 6550 operating in negative ESI mode. In an effort to detect dolichol we also purchased a dolichol standard (Avanti) but were unable to detect this by LC-MS. Metabolomics data have been deposited at Metabolomics workbench [59], study ST001188 (http://dx.doi.org/10.21228/M88M3Q).

### Immunoblot analysis

Synchronised ring-stage parasites (prepared by sorbitol treatment described as per *P*. *falciparum* culture) were treated with 50 μM indolmycin (BioAustralis Fine Chemicals) or dimethyl sulfoxide vehicle control for 30 hrs. The medium was replaced 30 hrs post drug administration, supplementing with the polyprenol rescue compound GGOH (5 μM) (Sigma Aldrich, G3278) as indicated. Trophozoites (5–10% parasitaemia) were collected at approximately 72–78 hrs post drug administration by lysis with 0.05% (w/v) saponin. Cells were then pelleted by centrifugation, washed with PBS, and then resuspended in Laemmli Sample Buffer (Bio-RAD) with 2.5% (v/v) 2-Mercaptoethanol and complete EDTA-free Protease Inhibitor (Roche). Samples were then either snap-frozen in liquid nitrogen and stored at -80°C or loaded directly onto Mini-PROTEAN™ TGX Precast Gels (Bio-RAD) in Tris-glycine buffer. Following electrophoresis at 180 Volts for 35 min, proteins were transferred to nitrocellulose membranes using the iBlot 2 western transfer system (Thermo Fisher Scientific).

PVDF membranes with bound protein were then blocked in 5% (w/v) skim milk in PBS, incubated with rabbit anti-farnesyl polyclonal antibody at 1:1,000 dilution (Life Technologies, PA1-12554) or mouse anti-BiP at 1:1,000 [60]. Following multiple washes with PBS-tween, horseradish peroxidase (HRP)-conjugated goat anti-rabbit IgG (PerkinElmer) or HRP-conjugated rabbit anti-mouse IgG (Promega) at 1:10,000 dilution was applied to membranes. Enhanced chemiluminescent detection was performed with SuperSignal® West Pico/Femto Sensitivity Substrate (Thermo Fisher Scientific) and analysed using the ChemiDoc™ Imaging System (Bio-RAD).

### Fluorescein dextran uptake assay

Using a modified version of the methodology described in Frankland and colleagues [35] and Baker and colleagues [36], packed RBCs were lysed with ice-cold lysis buffer (5 mM Na phosphate, 1 mM ATP; pH 7.5) containing 50 μM F-dextran (Life Technologies, D1820), and then incubated for 10 min at 4°C. The cells were then re-sealed by adding NaCl to 150 mM and incubating them for 45 min at 37°C. The re-sealed RBCs, loaded with F-dextran, were then washed in complete media (see above, *P*. *falciparum* culture) and stored at 4°C for up to one week. Internalisation assays were commenced by inoculating the preloaded RBCs with enriched schizonts (prepared as per *P*. *falciparum* culture), treating the newly invade ring-stage parasites with 50 μM indolmycin (BioAustralis Fine Chemicals) or dimethyl sulfoxide vehicle control for 30 hrs. The medium was replaced 30 hrs post drug administration, supplementing with the polyprenol rescue compound GGOH (5 μM) (Sigma Aldrich) as indicated. Trophozoites were collected at approximately 72–78 hrs post drug administration for live-cell microscopy. The iRBCs were washed for 5 min in 1 μg/mL Hoescht 33342 (Life Technologies) and mounted to directly to slides. Microscopy was performed using the DeltaVision Elite™ Widefield deconvolution (GE Healthcare) imaging platform. ImageJ (v1.51n) [61] was used to merge multiple microscopy channels. GraphPad Prism (V. 7.01) was used to plot data.

### Fluorescent microscopy

Fluorescent imaging of cells was performed either by live microscopy or indirect immunofluorescence assay. For live cell microscopy: iRBCs were collected, stained with 1 μg/mL Hoechst 33342 (Life Technologies), and mounted directly to glass slides. For indirect immunofluorescence assay: glass coverslips were pre-coated with 0.1 mg/mL pHAE (erythroagglutinating phytohemagglutinin) (Sigma Aldrich), and iRBCs were applied at 5% haematocrit in PBS to form a monolayer. The cells were then fixed on the coverslip with 2% (w/v) paraformaldehyde and 0.006% (w/v) glutaraldehyde for 20 min, permeabilised with 0.1% (v/v) Triton X-100 for 10 min, and then incubated with primary antibodies: 1:300 rabbit anti-GAP45 [41], 1:1,000 rabbit anti-REX1 [62] in 3% (w/v) BSA for 2 hrs. Following washing, Alexa Fluor 488 and 594-conjugated (Thermo Fisher scientific) anti-rabbit or anti-mouse secondary antibodies in 3% (w/v) BSA were applied to cells for 1 hr, washed with 300 nM DAPI (Sigma Aldrich), and mounted to slides with ProLong® Gold Antifade (Thermo Fisher scientific). Microscopy was performed using the DeltaVision Elite™ Widefield deconvolution (GE Healthcare) imaging platform. ImageJ (v1.51n) [61] was used to merge multiple microscopy channels.

### GFP-Rab5a localisation

Endogenously tagged GFP-2xFKBP-Rab5a [39] parasites were maintained in O+ RBCs as described [56] under selection with 4 nM WR99210 (Jacobus Pharmaceuticals). Parasite cultures were synchronized to 0–5 hpi and parental cultures were split into two dishes. Cells were exposed to 5 μM clindamycin (Ratiopharm) or vehicle (dH_2_O) for 35 hrs. Parasites were washed three times in complete RPMI medium (see above, *P*. *falciparum* culture), incubated for a further 51 hrs until 86–91 hpi and subsequently prepared for imaging.

### Imaging and analysis of GFP-Rab5a cell line

All live cell images of the GFP-Rab5a cell line haven been taken with a Carl Zeiss Axio Imager A1 and imaging was performed as described [63]. A 100×/1.4–numerical aperture lens combined with a Hamamatsu Orca C4742-95 camera was used. Parasite cultures were stained with 1 μg/mL DAPI for 15 min before imaging. Exposure time was identical for all acquired images on the GFP channel. Parasites to be analysed were selected from DIC images. The corresponding GFP image were loaded into ImageJ (v1.51n) [61]. Setting to be recorded were max grey value and mean grey value. The background fluorescence of the images as well as the maximum fluorescent intensity of the brightest focus within the parasite and the background fluorescence of each parasite (excluding GFP foci) were measured. Image background fluorescence intensity was subtracted from the latter two values and the ratio was calculated. For plotting of data GraphPad Prism (V. 7.01) was used. Representative images were processed with Corel Photo-Paint x6 by adjusting brightness and intensity (V. 16.4.1.1281).

### 3D block-face scanning electron microscopy

Synchronised 0–4 hpi ring-stage parasites (prepared as per *P*. *falciparum* culture) were treated with 50 μM indolmycin (BioAustralis Fine Chemicals) or dimethyl sulfoxide vehicle control for 30 hrs. Media was replaced 30 hrs post drug administration, and where indicated, cultures were supplemented with the polyprenol rescue compound GGOH (5 μM) (Sigma Aldrich). Trophozoites (5–10% parasitaemia) were collected at approximately 72–78 hrs post drug administration (equivalent to 28–34 hpi in the second IDC) by magnetic separation (as described above, *P*. *falciparum* culture) and fixed with 2.5% (w/v) glutaraldehyde in 0.1 M sodium cacodylate for at least 2 hrs at 4°C. Cells were washed in 0.175 M sodium cacodylate and then incubated en bloc in 1% (w/v) low-melt agarose with 1.5% (w/v) potassium ferrocyanide and 2% (w/v) osmium tetroxide in 0.15 M sodium cacodylate for 1 hr at 4°C. Cells were washed in Milli-Q® water and then incubated with 1% (w/v) thiocarbohydrazide for 20 min. Following further washes, cells were incubated with 2% (w/v) osmium textroxide for 30 min, 2% (w/v) uranyl acetate overnight at 4°C, and then Walton’s lead aspartate for 30 min at 60°C. Supernatant was then removed, samples were washed thoroughly with Milli-Q water before gradually dehydrated in ethanol, then acetone, and finally infiltrated with Procure 812 resin (ProSciTech). Samples were then polymerised by curing at 60°C for at least 24 hrs. Each polymerised block-face was trimmed to 1 mm^3^, and then serially sectioned (50 nm) and imaged using the Teneo Volume Scope (FEI) at 3kV. Image processing was performed using the IMOD software package (V. 4.9) [64]. For plotting of data GraphPad Prism (V. 7.01) was used.

### Dose-response assay

Synchronised ring-stage 3D7 parasites (prepared by sorbitol treatment described as per *P*. *falciparum* culture), or yDHODH parasites [65] maintained under selection with 5 nM WR99210 (Jacobus Pharmaceuticals), were set up in triplicate within V-bottom 96-well plates (1% haematocrit, 1% parasitaemia: 48 hrs; 0.1% parasitaemia: 96 hrs; 0.01% parasitaemia: 144 hrs) and treated with varying concentrations of indolmycin (BioAustralis Fine Chemicals), clindamycin hydrochloride (Sigma Aldrich), and chloramphenicol (Sigma Aldrich), prepared by serial dilution in complete medium (See above, *P*. *falciparum* culture). Equivalent iRBC wells were set up in complete medium only and positive-KILL controls were initiated in triplicate using these additional wells by adding 200 nM dihydroartemisinin (Sigma Aldrich) to ring-stage parasites 48 hrs prior to assaying growth inhibition. Complete medium was replaced after each IDC, and rescue compounds GGOH (Sigma Aldrich, G3278), FOH (Sigma Aldrich, F203), and IPP were added at varying concentration in complete medium for the cycles indicated. Growth inhibitions were analysed at 48, 96, and 144 hrs as indicated using SYBR Green assay as previously described [66], briefly RBC pellets were lysed and incubated with SYBR Green I (Thermo Fisher Scientific) for 1 hr and analysed using FLUOstar Omega plate reader (BMG Labtech). Percentage survival was determined by normalising the SYBR green signal of the treated conditions to the untreated controls, and then subtracting the background signal derived from the positive-KILL controls. EC50s given here are the concentration of compound required to inhibit 50% of this normalised SYBR green signal. Corrected values were plotted by XY scatter using GraphPad Prism (V. 7.01) and EC_50_ values were attained from the dose-response curves.

### Osmotic fragility assay

The osmotic fragility of indolmycin treated iRBCs were analysed using a protocol modified from Lew, Tiffert and Ginsburg [44], and Dennis and colleagues [46]. Synchronised 0–4 hpi ring-stage parasites (prepared as per *P*. *falciparum* culture) were treated with 50 μM indolmycin (BioAustralis Fine Chemicals) or dimethyl sulfoxide vehicle control for 30 hrs. Media was replaced 30 hrs post drug administration, and where indicated, cultures were supplemented with the polyprenol rescue compound GGOH (5 μM) (Sigma Aldrich). Trophozoites (2% haematocrit, 5–10% parasitaemia) were enriched at approximately 72–78 hrs post drug administration (equivalent to 28–34 hpi in the second IDC) by magnetic separation (as described above, *P*. *falciparum* culture). iRBC were resuspended in 400 μL of solution A (150 mM NaCl, 2 mM HEPES-Na, pH 7.4, relative tonicity (RT) = 1:300 mOsm). In a V-bottom 96-well plate, 200 μL of varying saline-tonic solutions were prepared in triplicate by diluting Solution A with Solution B (2 mM HEPES-Na, pH 7.4, RT = 0.04; 12 mOsm) to produce a range of saline tonic-solutions with RTs of 0.04 to 1. 10 μL of enriched iRBCs were then aliquoted into each tonic solution and incubated at room temperature for 10 min. Plates were then centrifuged at 1,200 g for 10 min and 175 μL of supernatants were transferred to a flat-bottom 96-well plate for analysis as described in Dennis and colleagues [46] using a FLUOstar Omega plate reader (BMG Labtech). GraphPad Prism (V. 7.01) was used to plot data.

## Supporting information

S1 Fig Dose-response curve to polyprenol compounds GGOH and FOH. SYBR-Green susceptibility assay determined 48 hrs post polyprenol treatment. Concentrations of GGOH greater than 20 μM and concentrations of FOH greater than 30 μM inhibit *P*. *falciparum* intraerythrocytic growth. Data are presented as the average of one experiment ± SD. See S2 Data for numerical data underlying figure. FOH, farnesol; GGOH, geranylgeraniol.
(TIFF)

S2 Fig Polyprenol supplementation with GGOH but not FOH protects parasites from the delayed death effect of apicoplast inhibitors. (A) Dose-response curve from SYBR-Green susceptibility assay determined 120 hrs post chloramphenicol treatment, with varying concentrations of GGOH supplemented as indicated. Data are presented as the means of three independent experiments ± SD. See S2 Data for numerical data underlying figure. (B) Dose-response curve from SYBR-Green susceptibility assay determined 120 hrs post chloramphenicol treatment, with GGOH (5 μM), FOH (5 μM), or GGOH (5 μM) plus FOH (5 μM) supplementation as indicated. Inhibition at 120 hrs is rescued by 5 μM GGOH but not 5 FOH or a combination of the two polyprenol compounds. Data are presented as the means of two independent experiments ± SD. See S2 Data for numerical data underlying figure. FOH, farnesol; GGOH, geranylgeraniol.
(TIF)

S3 Fig Polyprenol supplementation with geranylgeraniol protects parasites from the effect of clindamycin and chloramphenicol in the second IDC after treatment. (A) Dose-response curve from SYBR-Green susceptibility assay determined 48, 96, and 144 hrs post clindamycin treatment, with polyprenol (5 μM GGOH) supplementation as indicated. Clindamycin causes a delayed-death effect (inhibition at 96 but not 48 hrs) that is rescued by GGOH. However, inhibition at 144 hrs is not recued with GGOH supplementation. Data are presented as the means of three independent experiments ± SEM. See S2 Data for numerical data underlying figure. (B) Dose-response curve from SYBR-Green susceptibility assay determined 48, 96, and 144 hrs post chloramphenicol treatment, with polyprenol (5 μM GGOH) supplementation as indicated. Chloramphenicol causes a delayed-death effect (inhibition at 96 but not 48 hrs) that is rescued by GGOH. However, inhibition at 144 hrs is not recued with GGOH supplementation. Data are presented as the means of three independent experiments ± SEM. See S2 Data for numerical data underlying figure. GGOH, geranylgeraniol; IDC, intraerythrocytic development cycle.
(TIFF)

S4 Fig Aberrant morphology of DV in delayed-death parasites. Synchronised ring-stage parasites were treated with indolmycin (50 μM), with and without polyprenol rescue (5 μM GGOH) as indicated. Enriched trophozoite-stage parasites were collected for reduced osmium fixation 72–78 hrs post drug administration (equivalent to 28–32 hpi in the second IDC after treatment). Representative images (top-down and cross-sectional), from TEM of each condition: untreated, indolmycin treated, and indolmycin treated with polyprenol rescue. Structures indicated are N, DV, and CI. Scale bar = 1 μm. CI, cytostomal invagination; DV, digestive vacuole; GGOH, geranylgeraniol; IDC; intraerythrocytic developmental cycle; N, nucleus; TEM, transmission electron microscopy.
(TIFF)

S5 Fig Export of a PEXEL-negative protein is not affected by treatment with clindamycin or indolmycin. Representative immunofluorescence images of untreated, indolmycin- and clindamycin-treated parasites during the second IDC following treatment. Parasites were labelled with antisera (1:1,000) recognising REX1 (1:1,000). The exported protein REX1 localises to the RBC in indolmycin- and clindamycin-treated parasites equivalent to untreated. REX1, green signal; DAPI: parasite nuclei, blue signal; merge of green and blue signal. Scale bar = 5 μm. BF, bright field; DAPI, 4′,6-diamidino-2-phenylindole; IDC, intraerythrocytic developmental cycle; PEXEL, protein export elements; REX1, ring-exported protein 1.
(TIFF)

S1 Movie Z-stack from serial block-face scanning electron microscopy of delayed death parasites. Enriched trophozoite-stage parasites were collected for reduced osmium fixation 72–78 hrs post drug administration (equivalent to 28–32 hpi in the second IDC after treatment). Samples were imaged by SEM and subsequently trimmed 50 nM by automated diamond knife before reimaging. Imaging was repeated hundreds of times and parasite structures were manually traced for IMOD analysis. IDC, intraerythrocytic developmental cycle.
(AVI)

S2 Movie Serial block-face scanning electron microscopy of delayed death iRBC ZOOM. 3D-rendered iRBCs. Enriched trophozoite-stage parasites were collected for reduced osmium fixation 72–78 hrs post drug administration (equivalent to 28–32 hpi in the second IDC after treatment). Compartments are RBC (white), parasite (blue), cytostomal invaginations (yellow), and digestive vacuole (red). Shown is an indolmycin treated (50 μM) iRBC with two nuclei, *n* = 1. Scale bar = 1 μm. IDC, intraerythrocytic developmental cycle; iRBC, infected red blood cell.
(MOV)

S3 Movie Serial block-face scanning electron microscopy of untreated iRBCs. 3D-rendered iRBCs. Enriched trophozoite-stage parasites were collected for reduced osmium fixation 72–78 hrs post drug administration (equivalent to 28–32 hpi in the second IDC after treatment). Compartments are RBC (white), parasite (blue), CIs (yellow), and DV (red). Shown are untreated iRBCs with two nuclei, *n* = 7. CI, cytostomal invagination; DV, digestive vacuole; IDC, intraerythrocytic developmental cycle; iRBC, infected red blood cell.
(MOV)

S4 Movie Serial block-face scanning electron microscopy of delayed death iRBCs. 3D-rendered iRBCs. Enriched trophozoite-stage parasites were collected for reduced osmium fixation 72–78 hrs post drug administration (equivalent to 28–32 hpi in the second IDC after treatment). Compartments are RBC (white), parasite (blue), cytostomal invaginations (yellow), and digestive vacuole (red). Shown are indolmycin treated (50 μM) iRBCs with two nuclei, *n* = 7. IDC, intraerythrocytic developmental cycle; iRBC, infected red blood cell.
(MOV)

S5 Movie Serial block-face scanning electron microscopy of polyprenol rescued delayed-death iRBCs. 3D-rendered iRBCs. Enriched trophozoite-stage parasites were collected for reduced osmium fixation 72–78 hrs post drug administration (equivalent to 28–32 hpi in the second IDC after treatment). Compartments are iRBC (white), parasite (blue), CIs (yellow), and DV (red). Shown are indolmycin treated (50 μM) with GGOH supplementation (5 μM) iRBCs with two nuclei, *n* = 5. CI, cytostomal invagination; DV, digestive vacuole; IDC, intraerythrocytic developmental cycle; iRBC, infected red blood cell.
(MOV)

S1 Data Delayed-death metabolite time course. *P*. *falciparum*-infected cultures were collected for LC-MS metabolite detection across the first and second IDC following indolmycin treatment in comparison to an untreated control. IDC, intraerythrocytic developmental cycle; LC-MS, liquid chromatography mass spectrometry.
(XLSX)

S2 Data Summary data of numerical values that underlie quantitative analyses presented in the figures. (XLSX)

## Abbreviations

CI: cytostomal invagination
DAPI: 4′,6-diamidino-2-phenylindole
DOXP: 1-deoxy-xylulose-5-phosphate
DV: digestive vacuole
FASII: type II fatty acid synthesis
F-dextran: fluorescein dextran
FOH: farnesol
FPP: farnesyl pyrophosphate
GFP: green fluorescent protein
GGOH: geranylgeraniol
GPI: glycosylphosphatidylinosito
GGPP: geranylgeranyl pyrophosphate
hpi: hours post invasion
IDC: intraerythrocytic developmental cycle
IMC: inner membrane complex
IPP: isopentenyl pyrophosphate
iRBC: infected red blood cell
LC-MS: liquid chromatography mass spectrometry
MEP: 2-C-methyl-D-erythritol 4-phosphate
RBC: red blood cell
TrpRS^api^: apicoplast tryptophanyl-tRNA synthetase
yDHODH: yeast dihydroorotate dehydrogenase

## References

[1] Zhu G, Marchewka MJ, Keithly JS. *Cryptosporidium parvum* appears to lack a plastid genome. Microbiology. 2000;146: 315–321. 10.1099/00221287-146-2-315 10708370

[2] McFadden GI, Van Dooren GG. Evolution: Red algal genome affirms a common origin of all plastids. Curr Biol. 2004;14: 514–516. 10.1016/j.cub.2004.03.005 15242632

[3] Fast NM, Kissinger JC, Roos DS, Keeling PJ. Nuclear-encoded, plastid-targeted genes suggest a single common origin for apicomplexan and dinoflagellate plastids. Mol Biol Evol. 2001;18: 418–426. 10.1093/oxfordjournals.molbev.a003818 11230543

[4] Fichera ME, Roos DS. A plastid organelle as a drug target in apicomplexan parasites. Nature. 1997;390: 407–409. 10.1038/37132 9389481

[5] Ralph SA, D’Ombrain MC, McFadden GI. The apicoplast as an antimalarial drug target. Drug Resist Updat. 2001;4: 145–151. 10.1054/drup.2001.0205 11768328

[6] Dahl EL, Shock JL, Shenai BR, Gut J, DeRisi JL, Rosenthal PJ. Tetracyclines specifically target the apicoplast of the malaria parasite *Plasmodium falciparum*. Antimicrob Agents Chemother. 2006;50: 3124–3131. 10.1128/AAC.00394-06 16940111 PMC1563505

[7] Dahl EL, Rosenthal PJ. Multiple antibiotics exert delayed effects against the *Plasmodium falciparum* apicoplast. Antimicrob Agents Chemother. 2007;51: 3485–3490. 10.1128/AAC.00527-07 17698630 PMC2043295

[8] Camps M, Arrizabalaga G, Boothroyd J. An rRNA mutation identifies the apicoplast as the target for clindamycin in *Toxoplasma gondii*. Mol Microbiol. 2002;43: 1309–1318. 10.1046/j.1365-2958.2002.02825.x 11918815

[9] Goodman CD, Pasaje CFA, Kennedy K, McFadden GI, Ralph SA. Targeting protein translation in organelles of the Apicomplexa. Trends Parasitol. 2016;32: 953–965. 10.1016/j.pt.2016.09.011 27793563

[10] Pfefferkorn ER, Nothnagel RF, Borotz SE. Parasiticidal effect of clindamycin on *Toxoplasma gondii* grown in cultured cells and selection of a drug-resistant mutant. Antimicrob Agents Chemother. 1992;36: 1091–1096. 10.1128/aac.36.5.1091 1510399 PMC188841

[11] Rieckmann KH, Powell RD, McNamara J V., Willerson D, Lass L, Frischer H, et al Effects of tetracycline against chloroquine-resistant and chloroquine-sensitive *Plasmodium falciparum*. Am J Trop Med Hyg. 1971;20: 811–815. 10.4269/ajtmh.1971.20.811 4943475

[12] Goodman CD, Su V, McFadden GI. The effects of anti-bacterials on the malaria parasite *Plasmodium falciparum*. Mol Biochem Parasitol. 2007;152: 181–191. 10.1016/j.molbiopara.2007.01.005 17289168

[13] Friesen J, Silvie O, Putrianti ED, Hafalla JCR, Matuschewski K, Borrmann S. Natural immunization against malaria: causal prophylaxis with antibiotics. Sci Transl Med. 2010;2: 40ra49 10.1126/scitranslmed.3001058 20630856

[14] He CY, Shaw MK, Pletcher CH, Striepen B, Tilney LG, Roos DS. A plastid segregation defect in the protozoan parasite *Toxoplasma gondii*. EMBO J. 2001;20: 330–339. 10.1093/emboj/20.3.330 11157740 PMC133478

[15] Pfefferkorn ER, Borotz SE. Comparison of mutants of *Toxoplasma gondii* selected for resistance to azithromycin, spiramycin, or clindamycin. Antimicrob Agents Chemother. 1994;38: 31–37. 10.1128/aac.38.1.31 8141576 PMC284392

[16] Fichera ME, Bhopale MK, Roos DS. In vitro assays elucidate peculiar kinetics of clindamycin action against *Toxoplasma gondii*. Antimicrob Agents Chemother. 1995;39: 1530–1537. 10.1128/aac.39.7.1530 7492099 PMC162776

[17] Ralph SA, van Dooren GG, Waller RF, Crawford MJ, Fraunholz MJ, Foth BJ, et al Metabolic maps and functions of the *Plasmodium falciparum* apicoplast. Nat Rev Microbiol. 2004;2: 203–216. 10.1038/nrmicro843 15083156

[18] Yeh E, DeRisi JL. Chemical rescue of malaria parasites lacking an apicoplast defines organelle function in blood-stage *Plasmodium falciparum*. PLoS Biol. 2011;9 10.1371/journal.pbio.1001138 21912516 PMC3166167

[19] Amberg-Johnson K, Yeh E. Host cell metabolism contributes to delayed-death kinetics of apicoplast inhibitors in *Toxoplasma gondii*. Antimicrob Agents Chemother. 2019;63: e01646–18. 10.1128/AAC.01646-18 30455243 PMC6355570

[20] Jomaa H, Wiesner J, Sanderbrand S, Altincicek B, Weidemeyer C, Hintz M, et al Inhibitors of the nonmevalonate pathway of isoprenoid biosynthesis as antimalarial drugs. Science. 1999;285: 1573–1576. 10.1126/science.285.5433.1573 10477522

[21] Nair SC, Brooks CF, Goodman CD, Sturm A, McFadden GI, Sundriyal S, et al Apicoplast isoprenoid precursor synthesis and the molecular basis of fosmidomycin resistance in *Toxoplasma gondii*. J Exp Med. 2011;209: 1051–1051. 10.1084/jem.201100392095c PMC3135366 21690250

[22] Zhang M, Wang C, Otto TD, Oberstaller J, Liao X, Adapa SR, et al Uncovering the essential genes of the human malaria parasite *Plasmodium falciparum* by saturation mutagenesis. Science. 2018;360: eaap7847 10.1126/science.aap7847 29724925 PMC6360947

[23] Bushell E, Gomes AR, Sanderson T, Anar B, Girling G, Herd C, et al Functional Profiling of a *Plasmodium* Genome Reveals an Abundance of Essential Genes. Cell. 2017;170: 260–272.e8. 10.1016/j.cell.2017.06.030 28708996 PMC5509546

[24] Frénal K, Jacot D, Hammoudi PM, Graindorge A, MacO B, Soldati-Favre D. Myosin-dependent cell-cell communication controls synchronicity of division in acute and chronic stages of *Toxoplasma gondii*. Nat Commun. 2017;8 10.1038/ncomms15710 28593938 PMC5477499

[25] Guggisberg AM, Amthor RE, Odom AR. Isoprenoid Biosynthesis in *Plasmodium falciparum*. Eukaryot Cell. 2014;13: 1348–1359. 10.1128/EC.00160-14 25217461 PMC4248697

[26] Pasaje CFA, Cheung V, Kennedy K, Lim EE, Baell JB, Griffin MDW, et al Selective inhibition of apicoplast tryptophanyl-tRNA synthetase causes delayed death in *Plasmodium falciparum*. Sci Rep. 2016;6: 1–13. 10.1038/s41598-016-0001-8 27277538 PMC4899734

[27] Suazo KF, Schaber C, Palsuledesai CC, Odom John AR, Distefano MD. Global proteomic analysis of prenylated proteins in *Plasmodium falciparum* using an alkyne-modified isoprenoid analogue. Sci Rep. 2016;6: 1–11. 10.1038/s41598-016-0001-8 27924931 PMC5141570

[28] Gisselberg JE, Zhang L, Elias JE, Yeh E. The prenylated proteome of *Plasmodium falciparum* reveals pathogen-specific prenylation activity and drug mechanism-of-action. Mol Cell Proteomics. 2017;16: S54–S64. 10.1074/mcp.M116.064550 28040698 PMC5393391

[29] Elliott DA, McIntosh MT, Hosgood HD, Chen S, Zhang G, Baevova P, et al Four distinct pathways of hemoglobin uptake in the malaria parasite *Plasmodium falciparum*. Proc Natl Acad Sci. 2008;105: 2463–2468. 10.1073/pnas.0711067105 18263733 PMC2268159

[30] Howe R, Kelly M, Jimah J, Hodge D, Odoma AR. Isoprenoid biosynthesis inhibition disrupts Rab5 localization and food vacuolar integrity in *Plasmodium falciparum*. Eukaryot Cell. 2013;12: 215–223. 10.1128/EC.00073-12 23223036 PMC3571303

[31] Moura IC, Wunderlich G, Uhrig ML, Couto AS, Peres VJ, Katzin AM, et al Limonene arrests parasite development and inhibits isoprenylation of proteins in *Plasmodium falciparum*. Antimicrob Agents Chemother. 2001;45: 2553–2558. 10.1128/AAC.45.9.2553-2558.2001 11502528 PMC90691

[32] Chakrabarti D, Silva T Da, Barger J, Paquette S, Patel H, Patterson S, et al Protein farnesyltransferase and protein prenylation in *Plasmodium falciparum*. Biochemistry. 2002;277: 42066–42073. 10.1074/jbc.M202860200 12194969

[33] Zhang B, Watts KM, Hodge D, Kemp LM, Hunstad DA, Hicks LM, et al A second target of the antimalarial and antibacterial agent fosmidomycin revealed by cellular metabolic profiling. Biochemistry. 2011;50: 3570–3577. 10.1021/bi200113y 21438569 PMC3082593

[34] Uddin T, McFadden GI, Goodman CD. Validation of putative apicoplast-targeting drugs using a chemical supplementation assay in cultured human malaria parasites. Antimicrob Agents Chemother. American Society for Microbiology Journals; 2017;62: e01161–17. 10.1128/AAC.01161-17 29109165 PMC5740311

[35] Frankland S, Adisa A, Horrocks P, Taraschi TF, Schneider T, Elliott SR, et al Delivery of the malaria virulence protein PfEMP1 to the erythrocyte surface requires cholesterol-rich domains. Eukaryot Cell. 2006;5: 849–860. 10.1128/EC.5.5.849-860.2006 16682462 PMC1459682

[36] Bakar NA, Klonis N, Hanssen E, Chan C, Tilley L. Digestive-vacuole genesis and endocytic processes in the early intraerythrocytic stages of *Plasmodium falciparum*. J Cell Sci. 2010;123: 441–450. 10.1242/jcs.061499 20067995

[37] Jonscher E, Flemming S, Schmitt M, Sabitzki R, Reichard N, Birnbaum J, et al PfVPS45 Is required for host cell cytosol uptake by malaria blood stage parasites. Cell Host Microbe. 2019;25: 166–173.e5. 10.1016/j.chom.2018.11.010 30581113

[38] Kremer K, Kamin D, Rittweger E, Wilkes J, Flammer H, Mahler S, et al An overexpression screen of *Toxoplasma gondii* Rab-GTPases reveals distinct transport routes to the micronemes. PLoS Pathog. 2013;9 10.1371/journal.ppat.1003213 23505371 PMC3591302

[39] Birnbaum J, Flemming S, Reichard N, Soares AB, Mesén-Ramírez P, Jonscher E, et al A genetic system to study *Plasmodium falciparum* protein function. Nat Methods. 2017;14: 450–456. 10.1038/nmeth.4223 28288121

[40] Agop-Nersesian C, Naissant B, Rached F Ben, Rauch M, Kretzschmar A, Thiberge S, et al Rab11A-controlled assembly of the inner membrane complex is required for completion of apicomplexan cytokinesis. PLoS Pathog. 2009;5: e1000270 10.1371/journal.ppat.1000270 19165333 PMC2622761

[41] Baum J, Richard D, Healer J, Rug M, Krnajski Z, Gilberger TW, et al A conserved molecular motor drives cell invasion and gliding motility across malaria life cycle stages and other apicomplexan parasites. J Biol Chem. 2006;281: 5197–5208. 10.1074/jbc.M509807200 16321976

[42] Liu J, Istvan ES, Gluzman IY, Gross J, Goldberg DE. *Plasmodium falciparum* ensures its amino acid supply with multiple acquisition pathways and redundant proteolytic enzyme systems. Proc Natl Acad Sci. 2006;103: 8840–8845. 10.1073/pnas.0601876103 16731623 PMC1470969

[43] Krugliak M, Zhang J, Ginsburg H. Intraerythrocytic *Plasmodium falciparum* utilizes only a fraction of the amino acids derived from the digestion of host cell cytosol for the biosynthesis of its proteins. Mol Biochem Parasitol. 2002;119: 249–256. 10.1016/S0166-6851(01)00427-3 11814576

[44] Lew VL, Tiffert T, Ginsburg H. Excess hemoglobin digestion and the osmotic stability of *Plasmodium falciparum*-infected red blood cells. Blood. 2003;101: 4189–4194. 10.1182/blood-2002-08-2654 12531811

[45] Mauritz JMA, Esposito A, Ginsburg H, Kaminski CF, Tiffert T, Lew VL. The homeostasis of *Plasmodium falciparum*-infected red blood cells. PLoS Comput Biol. 2009;5 10.1371/journal.pcbi.1000339 19343220 PMC2659444

[46] Dennis ASM, Lehane AM, Ridgway MC, Holleran JP, Kirka K. Cell swelling induced by the antimalarial KAE609 (Cipargamin) and other PfATP4-associated antimalarials. Antimicrob Agents Chemother. 2018;62 10.1128/AAC.00087-18 29555632 PMC5971608

[47] Painter HJ, Morrisey JM, Mather MW, Vaidya AB. Specific role of mitochondrial electron transport in blood-stage *Plasmodium falciparum*. Nature. 2007;446: 88–91. 10.1038/nature05572 17330044

[48] Jackson KE, Pham JS, Kwek M, De Silva NS, Allen SM, Goodman CD, et al Dual targeting of aminoacyl-tRNA synthetases to the apicoplast and cytosol in *Plasmodium falciparum*. Int J Parasitol. 2012;42: 177–186. 10.1016/j.ijpara.2011.11.008 22222968

[49] Nallan L, Bauer KD, Bendale P, Rivas K, Yokoyama K, Hornéy CP, et al Protein farnesyltransferase inhibitors exhibit potent antimalarial activity. J Med Chem. 2005;48: 3704–3713. 10.1021/jm0491039 15916422

[50] Stenmark H. Rab GTPases as coordinators of vesicle traffic. Nat Rev Mol Cell Biol. 2009;10: 513–525. 10.1038/nrm2728 19603039

[51] Esposito A, Choimet JB, Skepper JN, Mauritz JMA, Lew VL, Kaminski CF, et al Quantitative imaging of human red blood cells infected with *Plasmodium falciparum*. Biophys J. 2010;99: 953–960. 10.1016/j.bpj.2010.04.065 20682274 PMC2913174

[52] Hanssen E, Knoechel C, Dearnley M, Dixon MWA, Le Gros M, Larabell C, et al Soft X-ray microscopy analysis of cell volume and hemoglobin content in erythrocytes infected with asexual and sexual stages of *Plasmodium falciparum*. J Struct Biol. 2012;177: 224–232. 10.1016/j.jsb.2011.09.003 21945653 PMC3349340

[53] Kirk K. Membrane Transport in the malaria-infected erythrocyte. Physiol Rev. 2017;81: 495–537. 10.1152/physrev.2001.81.2.495 11274338

[54] Staines HM, Ellory JC, Kirk K. Perturbation of the pump-leak balance for Na(+) and K(+) in malaria-infected erythrocytes. Am J Physiol Cell Physiol. 2001;280: C1576–87. 10.1152/ajpcell.2001.280.6.C1576 11350753

[55] Zarchin S, Krugliak M, Ginsburg H. Digestion of the host erythrocyte by malaria parasites is the primary target for quinolinecontaining antimalarials. Biochem Pharmacol. 1986;35: 2435–2442. 10.1016/0006-2952(86)90473-9 3524576

[56] Trager W, Jensen JB. Human malaria parasites in continuous culture. Science. 1976;193: 673–675. 10.1126/science.781840 781840

[57] Cobbold SA, Vaughan AM, Lewis IA, Painter HJ, Camargo N, Perlman DH, et al Kinetic flux profiling elucidates two independent acetyl-coa biosynthetic pathways in *Plasmodium falciparum*. J Biol Chem. 2013;288: 36338–36350. 10.1074/jbc.M113.503557 24163372 PMC3868748

[58] Cobbold SA, Chua HH, Nijagal B, Creek DJ, Ralph SA, McConville MJ. Metabolic dysregulation induced in *Plasmodium falciparum* by dihydroartemisinin and other front-line antimalarial drugs. J Infect Dis. 2016;213: 276–286. 10.1093/infdis/jiv372 26150544

[59] Sud M, Fahy E, Cotter D, Azam K, Vadivelu I, Burant C, et al Metabolomics Workbench: An international repository for metabolomics data and metadata, metabolite standards, protocols, tutorials and training, and analysis tools. Nucleic Acids Res. 2016;44: D463–D470. 10.1093/nar/gkv1042 26467476 PMC4702780

[60] Bridgford JL, Xie SC, Cobbold SA, Pasaje CFA, Herrmann S, Yang T, et al Artemisinin kills malaria parasites by damaging proteins and inhibiting the proteasome. Nat Commun. 2018;9 10.1038/s41467-017-01881-x 30228310 PMC6143634

[61] Schneider CA, Rasband WS, Eliceiri KW. NIH Image to ImageJ: 25 years of image analysis. Nat Methods. 2012;9: 671–675. 10.1038/nmeth.2089 22930834 PMC5554542

[62] Dixon MWA, Hawthorne PL, Spielmann T, Anderson KL, Trenholme KR, Gardiner DL. Targeting of the ring exported protein 1 to the maurer’s clefts is mediated by a two-phase process. Traffic. 2008;9: 1316–1326. 10.1111/j.1600-0854.2008.00768.x 18489703

[63] Grüring C, Spielmann T. Imaging of live malaria blood stage parasites. Methods Enzymol. 2012;506: 81–92. 10.1016/B978-0-12-391856-7.00029-9 22341220

[64] Kremer JR, Mastronarde DN, McIntosh JR. Computer visualization of three-dimensional image data using IMOD. J Struct Biol. 1996;116: 71–76. 10.1006/jsbi.1996.0013 8742726

[65] Dickerman BK, Elsworth B, Cobbold SA, Nie CQ, McConville MJ, Crabb BS, et al Identification of inhibitors that dually target the new permeability pathway and dihydroorotate dehydrogenase in the blood stage of *Plasmodium falciparum*. Sci Rep. 2016;6 10.1038/s41598-016-0015-2 27874068 PMC5118696

[66] Smilkstein M, Sriwilaijaroen N, Kelly JX, Wilairat P, Riscoe M. Simple and inexpensive fluorescence-based technique for high-throughput antimalarial drug screening. Antimicrob Agents Chemother. 2004;48: 1803–1806. 10.1128/AAC.48.5.1803-1806.2004 15105138 PMC400546

