# Supplementary figures and images for: Delayed death in the malaria parasite Plasmodium falciparum is caused by disruption of prenylation-dependent intracellular trafficking
Source: PLoS Biol. 2019 Jul 18;17(7):e3000376. doi: 10.1371/journal.pbio.3000376 (PMC6667170; doi:10.1371/journal.pbio.3000376)

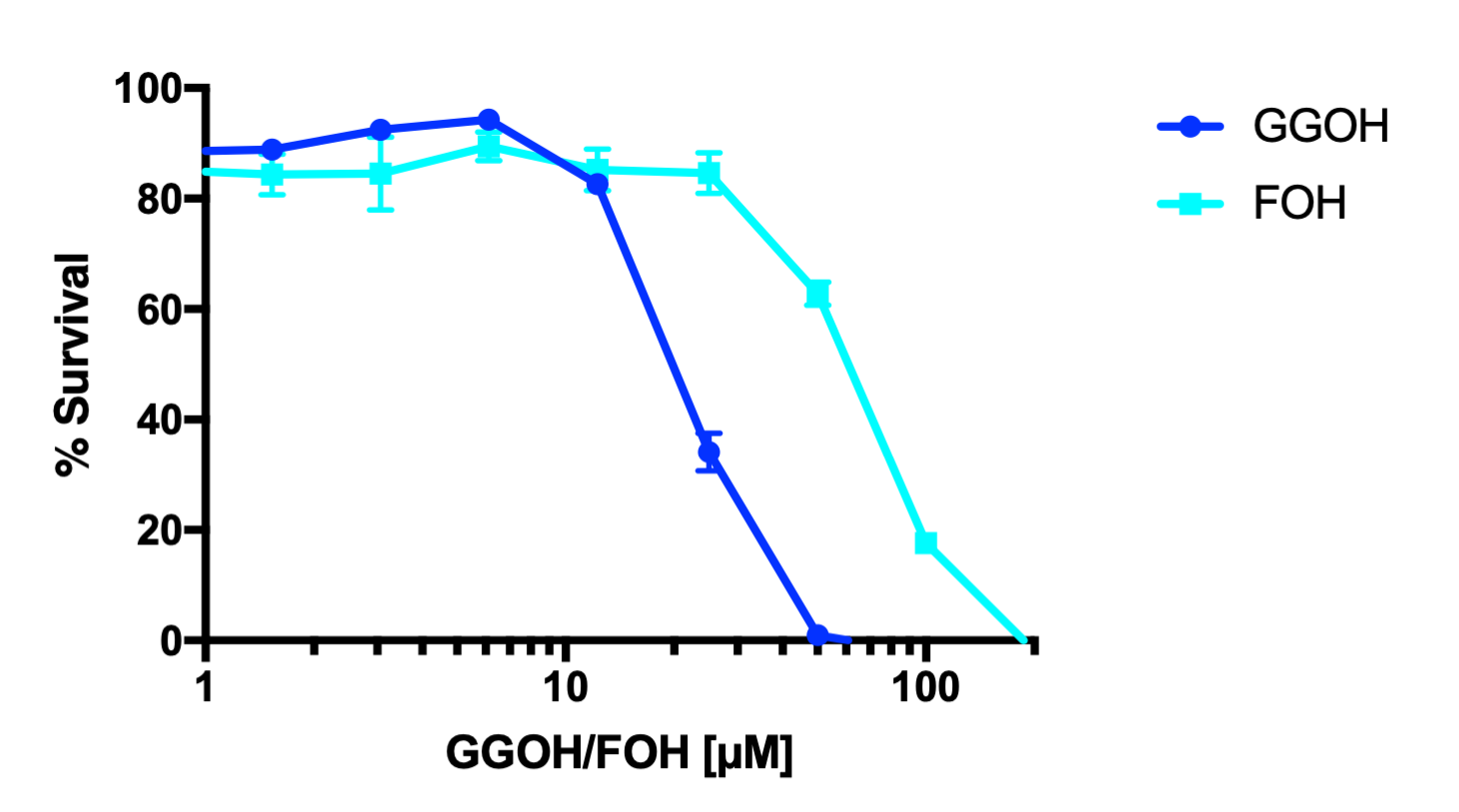

Supplement: S1 Fig — SYBR-Green susceptibility assay determined 48 hrs post polyprenol treatment. Concentrations of GGOH greater than 20 μM and concentrations of FOH greater than 30 μM inhibit P. falciparum intraerythrocytic growth. Data are presented as the average of one experiment ± SD. See S2 Data for numerical data underlying figure. FOH, farnesol; GGOH, geranylgeraniol. (TIFF) [file pbio.3000376.s001.tiff]

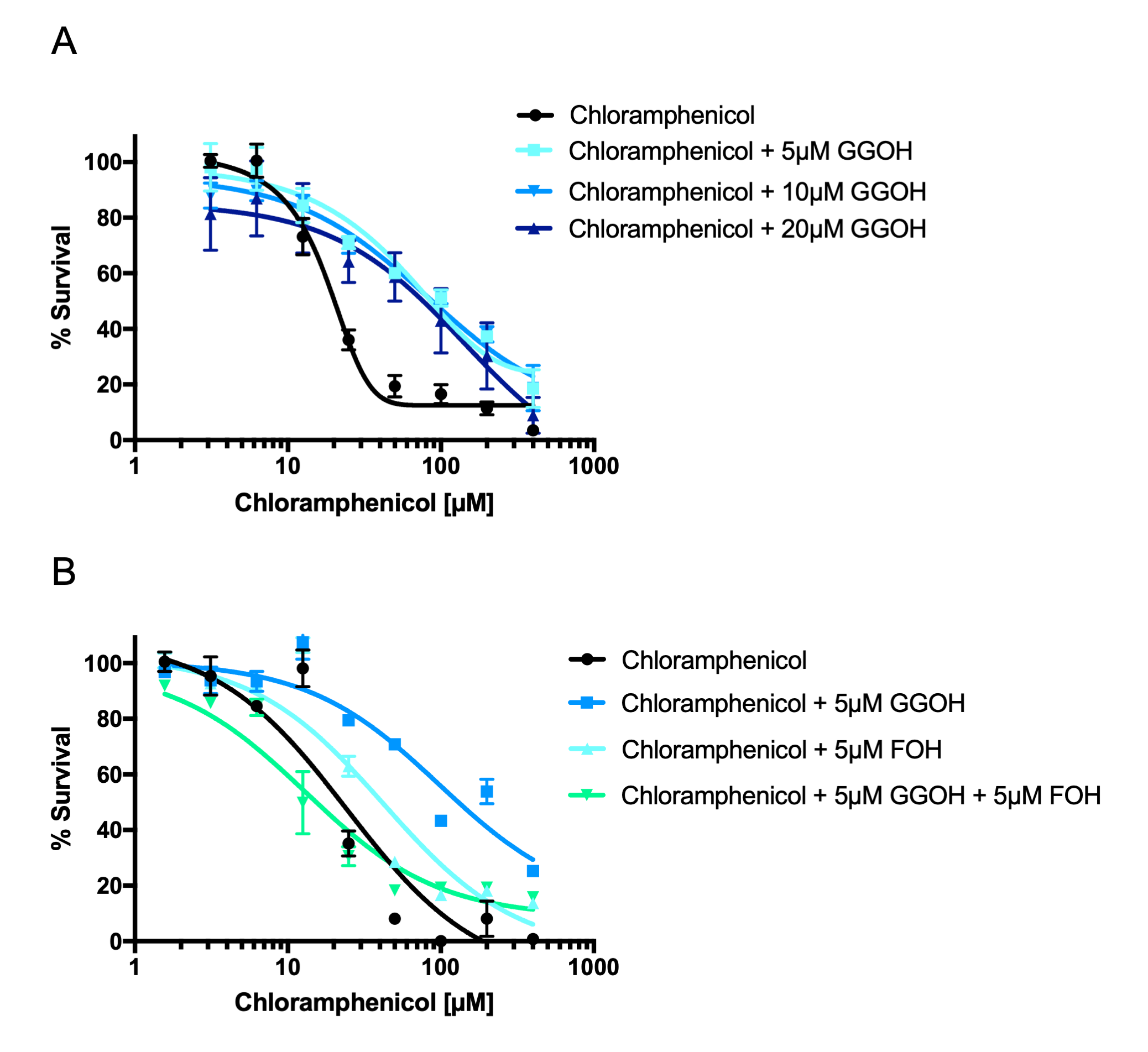

Supplement: S2 Fig — (A) Dose-response curve from SYBR-Green susceptibility assay determined 120 hrs post chloramphenicol treatment, with varying concentrations of GGOH supplemented as indicated. Data are presented as the means of three independent experiments ± SD. See S2 Data for numerical data underlying figure. (B) Dose-response curve from SYBR-Green susceptibility assay determined 120 hrs post chloramphenicol treatment, with GGOH (5 μM), FOH (5 μM), or GGOH (5 μM) plus FOH (5 μM) supplementation as indicated. Inhibition at 120 hrs is rescued by 5 μM GGOH but not 5 FOH or a combination of the two polyprenol compounds. Data are presented as the means of two independent experiments ± SD. See S2 Data for numerical data underlying figure. FOH, farnesol; GGOH, geranylgeraniol. (TIF) [file pbio.3000376.s002.tif]

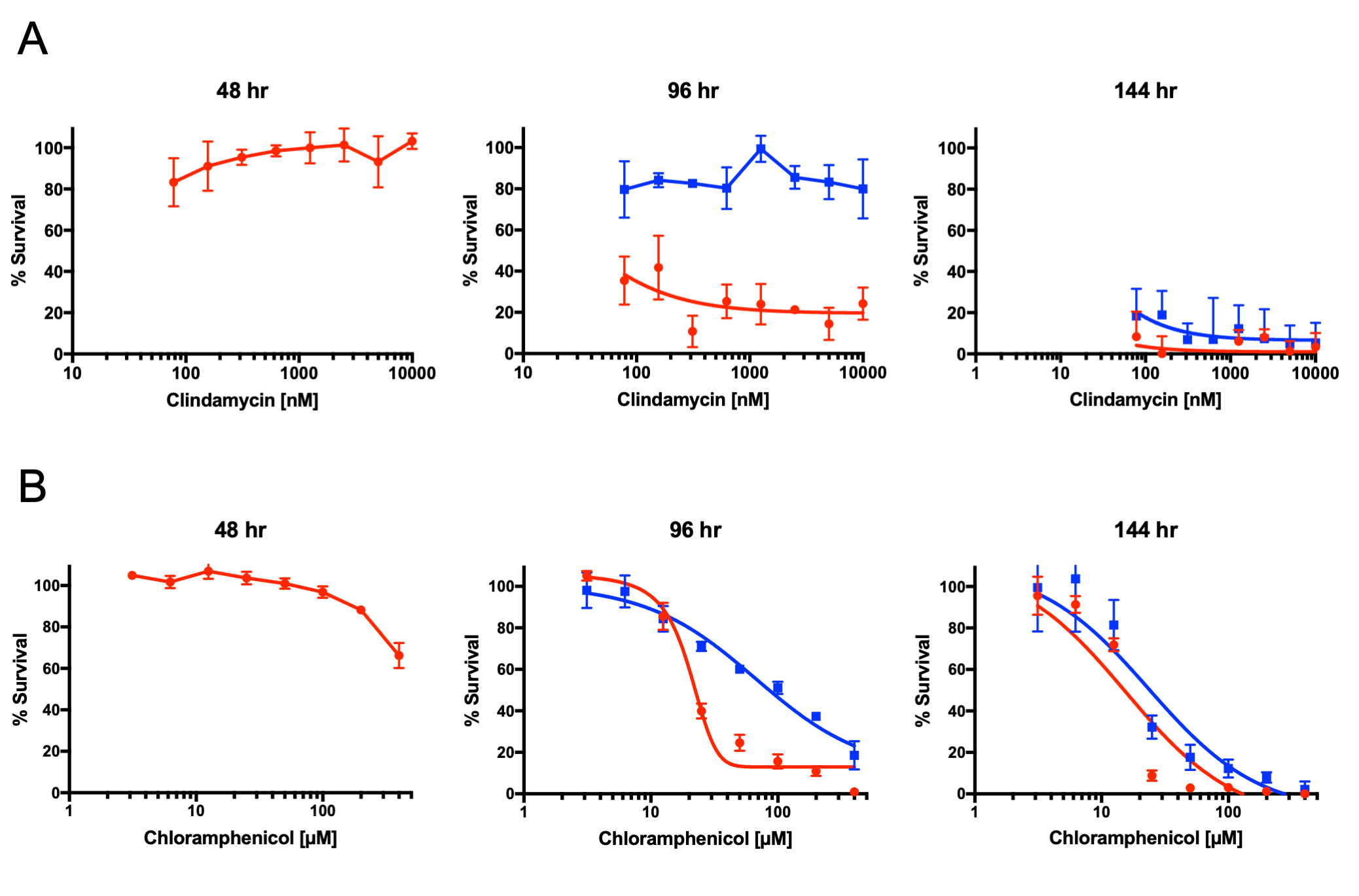

Supplement: S3 Fig — (A) Dose-response curve from SYBR-Green susceptibility assay determined 48, 96, and 144 hrs post clindamycin treatment, with polyprenol (5 μM GGOH) supplementation as indicated. Clindamycin causes a delayed-death effect (inhibition at 96 but not 48 hrs) that is rescued by GGOH. However, inhibition at 144 hrs is not recued with GGOH supplementation. Data are presented as the means of three independent experiments ± SEM. See S2 Data for numerical data underlying figure. (B) Dose-response curve from SYBR-Green susceptibility assay determined 48, 96, and 144 hrs post chloramphenicol treatment, with polyprenol (5 μM GGOH) supplementation as indicated. Chloramphenicol causes a delayed-death effect (inhibition at 96 but not 48 hrs) that is rescued by GGOH. However, inhibition at 144 hrs is not recued with GGOH supplementation. Data are presented as the means of three independent experiments ± SEM. See S2 Data for numerical data underlying figure. GGOH, geranylgeraniol; IDC, intraerythrocytic development cycle. (TIFF) [file pbio.3000376.s003.tiff]

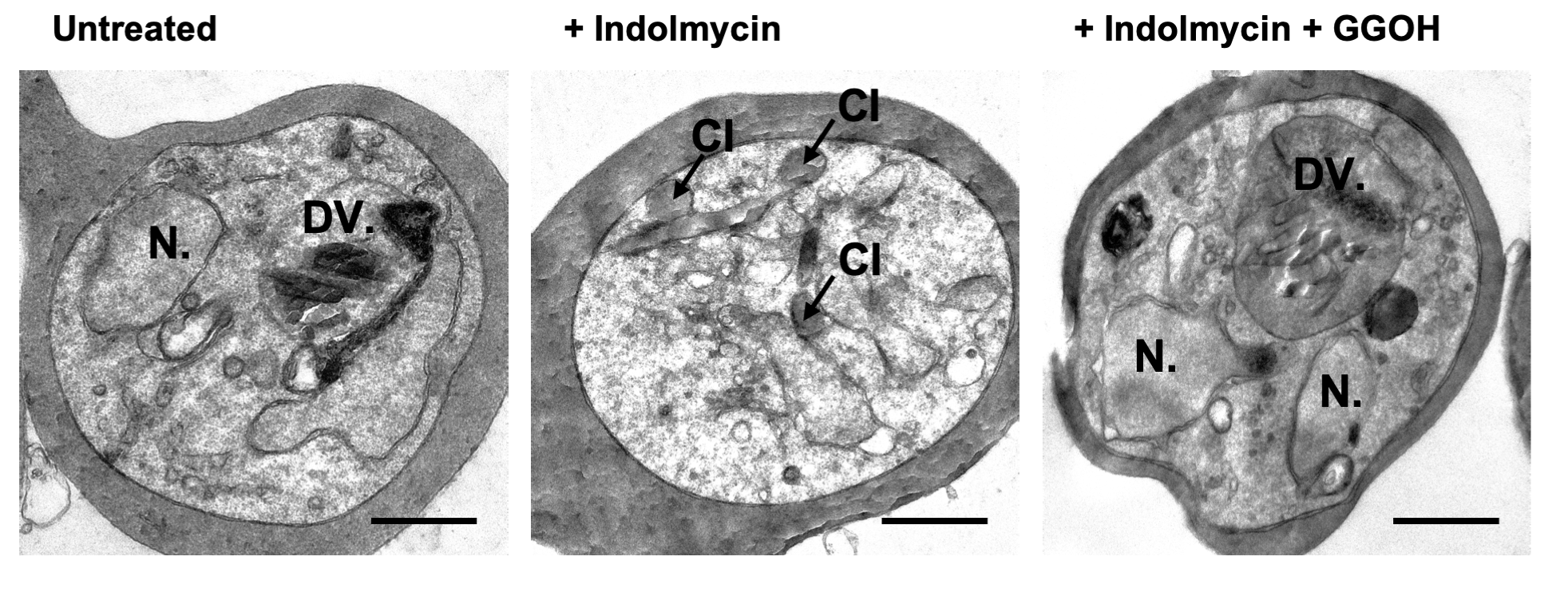

Supplement: S4 Fig — Synchronised ring-stage parasites were treated with indolmycin (50 μM), with and without polyprenol rescue (5 μM GGOH) as indicated. Enriched trophozoite-stage parasites were collected for reduced osmium fixation 72–78 hrs post drug administration (equivalent to 28–32 hpi in the second IDC after treatment). Representative images (top-down and cross-sectional), from TEM of each condition: untreated, indolmycin treated, and indolmycin treated with polyprenol rescue. Structures indicated are N, DV, and CI. Scale bar = 1 μm. CI, cytostomal invagination; DV, digestive vacuole; GGOH, geranylgeraniol; IDC; intraerythrocytic developmental cycle; N, nucleus; TEM, transmission electron microscopy. (TIFF) [file pbio.3000376.s004.tiff]

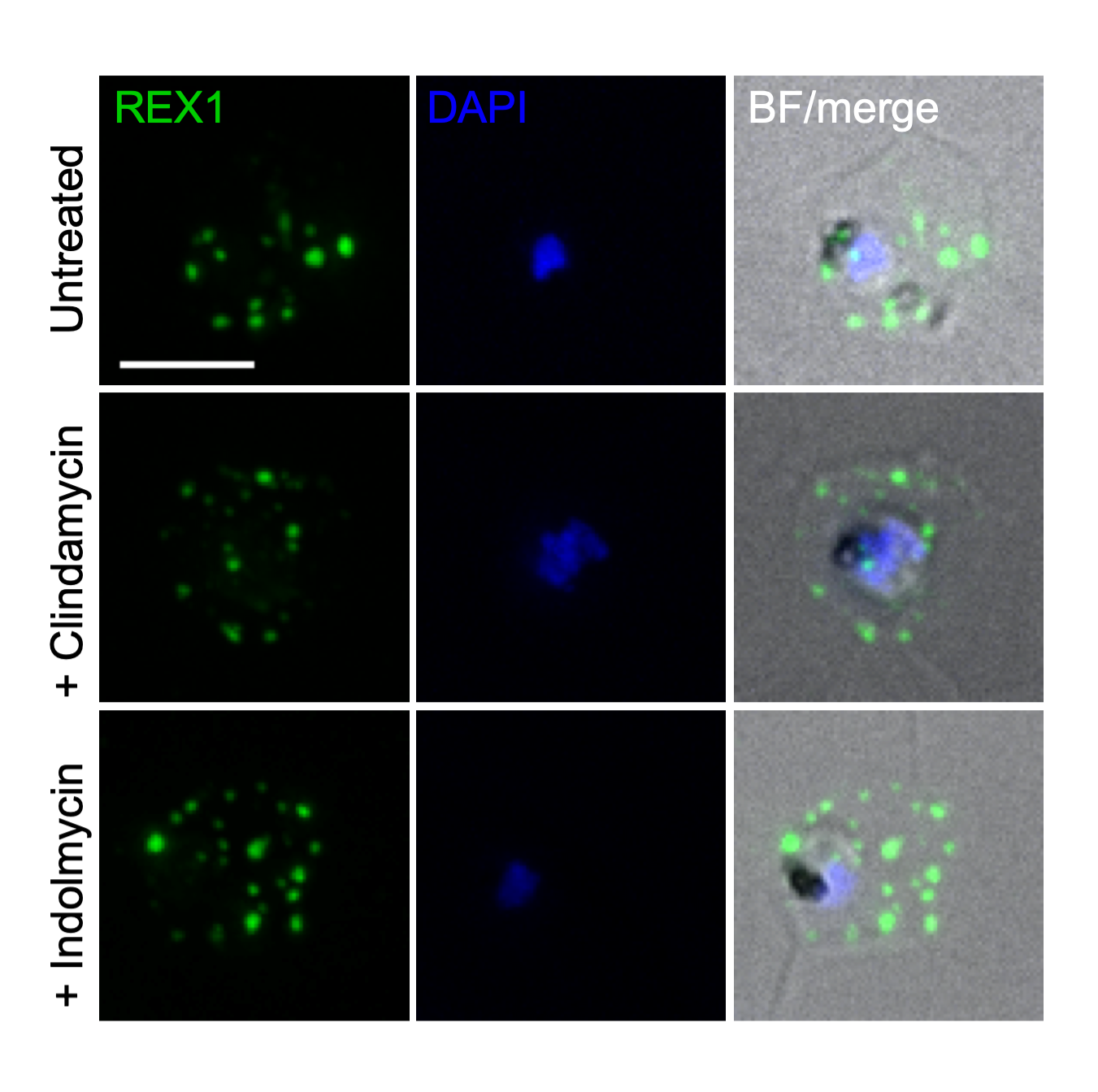

Supplement: S5 Fig — Representative immunofluorescence images of untreated, indolmycin- and clindamycin-treated parasites during the second IDC following treatment. Parasites were labelled with antisera (1:1,000) recognising REX1 (1:1,000). The exported protein REX1 localises to the RBC in indolmycin- and clindamycin-treated parasites equivalent to untreated. REX1, green signal; DAPI: parasite nuclei, blue signal; merge of green and blue signal. Scale bar = 5 μm. BF, bright field; DAPI, 4′,6-diamidino-2-phenylindole; IDC, intraerythrocytic developmental cycle; PEXEL, protein export elements; REX1, ring-exported protein 1. (TIFF) [file pbio.3000376.s005.tiff]
